# Supplementary material for: Tuning parameters for polygenic risk score methods using GWAS summary statistics from training data
Source: Nat Commun. 2024 Jan 2;15:24. doi: 10.1038/s41467-023-44009-0 (PMC10762162; doi:10.1038/s41467-023-44009-0)
Supplement: Supplementary file 1 — Supplementary Information [file 41467_2023_44009_MOESM1_ESM.pdf]

Tuning Parameters for Polygenic Risk Score Methods Using GWAS Summary Statistics from Training Data

Supplementary Notes

1 Supplementary Methods

1.1 Derivation for the posterior distribution of the allele frequency differences among independent SNPs

Property 1. *If* $X|Y \sim N_p(Y, \Sigma)$, $Y \sim N_p(Y_0, \Sigma_0)$, *Then*

$$\begin{aligned} &X \sim N_p(Y_0, \Sigma + \Sigma_0); \\ &Y|X \sim N_p(WY_0 + (I - W)X, (I - W)\Sigma), \end{aligned} \tag{1}$$

where $W = \Sigma(\Sigma_0 + \Sigma)^{-1}$.

The proof of this property can be found in Chapter 2 of (Bishop and Nasrabadi, 2006).

In our case, we have the following distributions for z-scores of independent SNPs:

$$\begin{aligned} &z_m|\beta_m \sim N(\frac{\beta_m}{s_m}, 1), \\ &\frac{\beta_m}{s_m} \sim (1 - \pi)\delta_0 + \pi N(0, \sigma^2). \end{aligned} \tag{2}$$

Based on Property 1, we can derive the marginal distribution of z_m:

$$z_m \sim (1 - \pi)N(0, 1) + \pi N(0, 1 + \sigma^2). \tag{3}$$

The posterior probability for $\beta_m = 0$ is

$$
\begin{aligned}
1 - h_m := & \Pr(\beta_m = 0 | z_m) \\
= & \frac{(1-\pi)\phi(z_m)}{(1-\pi)\phi(z_m) + \frac{\pi}{\sqrt{1+\sigma^2}}\phi(z_m/\sqrt{1+\sigma^2})}.
\end{aligned}
\tag{4}
$$

When β_m comes from the normal distribution component, we can derive the posterior distribution of β_m using Property 1. In summary, the distribution of $\frac{\beta_m}{s_m}|z_m$ is formulated as

$$
\frac{\beta_m}{s_m}|z_m \sim (1-h_m)\delta_0 + h_m N(\lambda z_m, \lambda), \tag{5}
$$

where $\lambda = \frac{1}{1+1/\sigma^2}$ is the shrinkage effect factor.

Due to the independence among the pre-selected SNPs, the allele frequency difference of SNP m is equal to the risk effect size, i.e., $\delta_m = \beta_m$. Hence we have

$$
\delta_m | z_m \sim (1-h_m)\delta_0 + h_m N(\lambda z_m s_m, \lambda s_m^2). \tag{6}
$$

1.2 Joint distribution of z-scores among dependent SNPs

In this section, we will prove the following property for the distribution of z-scores extracted from a GWAS of a disease:

Property 2. *For M SNPs, we use $\boldsymbol{\beta}$ to represent the underlying effects of changing allele frequencies between two groups. Then we have*

$$
\boldsymbol{z}|\boldsymbol{\beta} \sim N(\boldsymbol{R}\boldsymbol{S}^{-1}\boldsymbol{\beta}, \boldsymbol{R}) \tag{7}
$$

where $\boldsymbol{S}$ is a diagonal matrix with the diagonal elements encoding the standard errors of the observed allele frequency differences $\hat{f}_1 - \hat{f}_0$, and $\boldsymbol{R}$ is the LD matrix representing the pairwise correlations among SNP genotypes due to LD.

Proof. For diseases and other binary traits, a commonly used test statistic for binary traits is the frequency difference test, in which a z-score for SNP A is calculated based on the following formula:

$$
z_A = \frac{\hat{f}_{A1} - \hat{f}_{A0}}{s_A}, \tag{8}
$$

where $s_A = \sqrt{\frac{\hat{f}_{A1}(1-\hat{f}_{A1})}{2n_1} + \frac{\hat{f}_{A0}(1-\hat{f}_{A0})}{2n_0}}$ is the standard error of $\hat{f}_1 - \hat{f}_0$ based on the variance formula of a binomial distribution. Here we assume the potential allele frequencies among cases and controls under

an assumed condition that the SNP is independent of others are p_{A1} and p_{A0}, respectively. We define the true effect of SNP A as $\beta_A = p_{A1} - p_{A0}$. We use f_{A1} and f_{A0} denoting the expectations of observed allele frequencies among cases and controls, which are the allele frequencies marginalizing over other SNPs. We have

$$s_A \approx \sqrt{\frac{f_{A1}f_{a1}}{2n_1} + \frac{f_{A0}f_{a0}}{2n_0}} \approx \sqrt{(\frac{1}{2n_1} + \frac{1}{2n_0})f_A f_a}, \tag{9}$$

where $f_A = \frac{n_0 f_{A0} + n_1 f_{A1}}{n_0 + n_1}$ and $f_a = \frac{n_0 f_{a0} + n_1 f_{a1}}{n_0 + n_1}$ are the pooled allele frequencies of allele A/a among all samples under expectation. The table on the following page summarizes the notations we shall use here.

1. First, let's consider $Cov(\boldsymbol{z})$ under this setting. We will prove the covariance between test statistics of two SNPs is the same as that between the two genotypes:

$$Cov(\boldsymbol{z}) = \boldsymbol{R}. \tag{10}$$

We consider two SNPs, A and B, with alleles denoted as "Aa" and "Bb", respectively. We use "1" to denote case and "0" to denote control.

Based on the definition of z-scores, the covariance between z_A and z_B reads:

$$Cov(z_A, z_B) \approx \frac{1}{(\frac{1}{2n_0} + \frac{1}{2n_1})\sqrt{f_A f_a f_B f_b}}(Cov(\hat{f}_{A1}, \hat{f}_{B1}) + Cov(\hat{f}_{A0}, \hat{f}_{B0}) - Cov(\hat{f}_{A1}, \hat{f}_{B0}) - Cov(\hat{f}_{A0}, \hat{f}_{B1})). \tag{11}$$

Here,

$$\begin{aligned} Cov(\hat{f}_{A1}, \hat{f}_{B1}) &= \frac{1}{4n_1^2} Cov(n_{A1}, n_{B1}) \\ &= \frac{1}{4n_1^2} Cov(\sum_{i=1}^{2n_1} \mathbb{1}_{A1i}, \sum_{i=1}^{2n_1} \mathbb{1}_{B1i}) \\ &= \frac{1}{4n_1^2} \sum_{i=1}^{2n_1} Cov(\mathbb{1}_{A1i}, \mathbb{1}_{B1i}) \\ &= \frac{1}{4n_1^2} 2n_1 D_{AB} \\ &= \frac{D_{AB}}{2n_1}, \end{aligned} \tag{12}$$

Similarly, $Cov(\hat{f}_{A0}, \hat{f}_{B0}) = \frac{D_{AB}}{2n_0}$, $Cov(\hat{f}_{A1}, \hat{f}_{B0}) = 0$ and $Cov(\hat{f}_{A0}, \hat{f}_{B1}) = 0$.

notation	meaning
z_A	z-score of SNP A
$\hat{f}_{A1}/\hat{f}_{a1}$	Observed allele frequency of A/a in case group
$\hat{f}_{A0}/\hat{f}_{a0}$	Observed allele frequency of A/a in control group
$\hat{f}_{A}/\hat{f}_{a}$	Pooled observed allele frequency of A/a
p_{A1}/p_{a1}	Potential allele frequency of A/a in case group without influence from other SNPs
p_{A0}/p_{a0}	Potential allele frequency of A/a in control group without influence from other SNPs
p_A/p_a	Potential pooled allele frequency of A/a without influence from other SNPs
f_{A1}/f_{a1}	Expected allele frequency of A/a in case group
f_{A0}/f_{a0}	Expected allele frequency of A/a in control group
f_A/f_a	Pooled allele frequency of A/a under expectation
$\mathbb{1}_{A1i}/\mathbb{1}_{a1i}$	Indicator function that indicates whether the allele for the ith sample in case group is A/a
$\mathbb{1}_{A0i}/\mathbb{1}_{a0i}$	Indicator function that indicates whether the allele for the ith sample in control group is A/a
n_0/n_1	sample size for control/case group
D_{AB}	The coefficient of linkage disequilibrium between SNP A and B
r_{AB}	The correlation coefficient between SNP A and B

Supplementary Table 1: The table of notations.

Thus:

$$\begin{aligned} Cov(z_A, z_B) &= \frac{1}{(\frac{1}{2n_0} + \frac{1}{2n_1})\sqrt{f_A f_a f_B f_b}}(\frac{D_{AB}}{2n_1} + \frac{D_{AB}}{2n_0}) \\ &= \frac{D_{AB}}{\sqrt{f_A f_a f_B f_b}} \\ &= r_{AB} \,. \end{aligned} \tag{13}$$

This relationship is valid for any pair of SNPs.

2. Then let's consider $E(\boldsymbol{z})$ under this setting:

$$E(\boldsymbol{z}) = \boldsymbol{R}\boldsymbol{S}^{-1}\boldsymbol{\beta}, \tag{14}$$

where $\boldsymbol{S} = \text{diag}(se(\hat{\beta}_j))$ is a diagonal matrix storing the standard errors of $\hat{f}_1 - \hat{f}_0$.

We consider a so-called "linked SNP" A. First, we suppose A is linked with only one risk SNP B. Then, we will show the case that $s = 2$, thus A is linked with two risk SNPs B and C. In the end, we generalize the result to s risk SNPs, in other words $E(z_A)$ can be represented as a linear combination of $\frac{\beta_{c1}}{s_{c_1}}, \frac{\beta_{c2}}{s_{c_2}}, \ldots, \frac{\beta_{c_s}}{s_{c_s}}$, where $c_1, c_2, \ldots, c_s$ are indices of s risk SNPs.

(a) SNP A is linked with only one risk SNP B.

In this part, we will prove that: $E(z_A) = r_{AB}\frac{\beta_B}{s_B}$. Here we assume that the LD structure is consistent in a population and has nothing to do with whether a person belongs to the case or control group. Then we have

$$\begin{aligned} p_{A|B1} &\equiv P(A\big|B, case) \\ &= \frac{P(A\big|B, case)P(B\big|case)}{P(B\big|case)} \\ &= \frac{P(A\big|B)P(B\big|case)}{P(B\big|case)} \\ &= p_{A|B} = p_{A|B0} \,. \end{aligned} \tag{15}$$

Then,

$$
\begin{aligned}
\Delta f_A \equiv f_{A1} - f_{A0} &= (p_{A|B1}p_{B1} + p_{A|b1}p_{b1}) - (p_{A|B0}p_{B0} + p_{A|b0}p_{b0}) \\
&= p_{A|B}(p_{B1} - p_{B0}) + p_{A|b}(p_{b1} - p_{b0}) \\
&= p_{A|B}(p_{B1} - p_{B0}) + p_{A|b}((1 - p_{B1}) - (1 - p_{B0})) \\
&= (p_{A|B} - p_{A|b})(p_{B1} - p_{B0}) \\
&= (\frac{p_{AB}}{f_B} - \frac{p_{Ab}}{f_b})(p_{B1} - p_{B0}) \\
&= \frac{p_{AB}(p_{Ab} + p_{ab}) - p_{Ab}(p_{AB} + p_{aB})}{f_B f_b}\beta_B \\
&= \frac{p_{AB}p_{ab} - p_{Ab}p_{aB}}{f_B f_b}\beta_B \\
&= \frac{D_{AB}}{f_B f_b}\beta_B.
\end{aligned} \tag{16}
$$

Thus,

$$
E(z_A) = r_{AB}\frac{\beta_B}{s_B} \tag{17}
$$

(b) SNP A is linked with two independent causal SNPs B and C.

In this part, we will prove that: $E(z_A) = r_{AB}\frac{\beta_B}{s_B} + r_{AC}\frac{\beta_C}{s_C}$.

$$
\begin{aligned}
\Delta f_A = f_{A1} - f_{A0} &= (p_{A|BC1}p_{BC1} + p_{A|Bc1}p_{Bc1} + p_{A|bC1}p_{bC1} + p_{A|bc1}p_{bc1}) \\
&- (p_{A|BC0}p_{BC0} + p_{A|Bc0}p_{Bc0} + p_{A|bC0}p_{bC0} + p_{A|bc0}p_{bc0}) \\
&= p_{A|BC}(p_{BC1} - p_{BC0}) + p_{A|Bc}(p_{Bc1} - p_{Bc0}) \\
&+ p_{A|bC}(p_{bC1} - p_{bC0}) + p_{A|bc}(p_{bc1} - p_{bc0})\,.
\end{aligned} \tag{18}
$$

Consider the first term:

$$
\begin{aligned}
p_{A|BC} &= \frac{p_{ABC}}{p_{BC}} \\
&= \frac{p_{AB}p_{C|AB}}{f_B f_C} \\
&= \frac{p_{AB}p_{C|A}}{f_B f_C} \\
&= \frac{p_{AB}p_{AC}}{f_A f_B f_C}
\end{aligned} \tag{19}
$$

and

$$
\begin{aligned}
\Delta p_{BC} &= p_{BC1} - p_{BC0} \\
&= p_{B1}p_{C1} - p_{B0}p_{C0} \\
&= p_{B1}p_{C1} - p_{B1}p_{C0} + p_{B1}p_{C0} - p_{B0}p_{C0} \\
&= p_{B1}\beta_C + p_{C0}\beta_B \\
&\approx f_B\beta_C + f_C\beta_B\,.
\end{aligned}
\tag{20}
$$

Then,

$$
\begin{aligned}
\Delta f_A = f_{A1} - f_{A0} &= \frac{p_{AB}p_{AC}}{f_A f_B f_C}(f_B\beta_C + f_C\beta_B) + \frac{p_{AB}p_{Ac}}{f_A f_B f_c}(-f_B\beta_C + f_c\beta_B) \\
&+ \frac{p_{Ab}p_{AC}}{f_A f_b f_C}(f_b\beta_C - f_C\beta_B) + \frac{p_{Ab}p_{Ac}}{f_A f_b f_c}(-f_b\beta_C - f_c\beta_B) \\
&= (p_{A|C} - p_{A|c})\beta_C + (p_{A|B} - p_{A|b})\beta_B \\
&= \frac{D_{AC}}{f_C f_c}\beta_C + \frac{D_{AB}}{f_B f_b}\beta_B.
\end{aligned}
\tag{21}
$$

Thus, we have:

$$
E(z_A) = r_{AB}\frac{\beta_B}{s_B} + r_{AC}\frac{\beta_C}{s_C} \tag{22}
$$

(c) SNP A is linked with s independent risk SNPs: B_1, B_2, ..., B_s

To clarify our proof, we use $B_i (i = 1, 2, \cdots, s)$ to denote the major allele of SNP B_i and b_i is the minor allele of SNP B_i. In the proof below,

$$
\sum_{B_1, B_2, \cdots, B_s}
$$

means looping through all possible combinations of alleles for SNPs $B_1, B_2, \cdots, B_s$.

Then, we have:

$$
\Delta f_A = \sum_{B_1, B_2, \cdots, B_s} p_{A|B_1, B_2, \cdots, B_s} \Delta p_{B_1, B_2, \cdots, B_s}\,, \tag{23}
$$

where

$$
p_{A|B_1, B_2, \cdot, B_s} = \frac{p_{AB_1}p_{AB_2}\cdots p_{AB_s}}{f_A^{s-1} f_{B_1} f_{B_2}\cdots f_{B_s}}, \tag{24}
$$

$$
\Delta p_{B_1, B_2, \cdots, B_s} = \sum_{i=1}^{s} \prod_{j=1 j\neq i}^{s} f_{B_j}\beta_{B_i}. \tag{25}
$$

Therefore,

$$
\begin{aligned}
\Delta f_A &= \sum_{B_1,B_2,\cdots,B_s} \frac{p_{AB_1}p_{AB_2}\cdots p_{AB_s}}{f_A^{s-1} f_{B_1} f_{B_2}\cdots f_{B_s}} \sum_{i=1}^{s}(\prod_{j=1 j\neq i}^{s} f_{B_j})\beta_{B_i} \\
&= \sum_{B_1,B_2,\cdots,B_s} \sum_{i=1}^{s} \frac{p_{AB_1}p_{AB_2}\cdots p_{AB_s}}{f_A^{s-1} f_{B_1} f_{B_2}\cdots f_{B_s}} (\prod_{j=1 j\neq i}^{s} f_{B_j})\beta_{B_i} \\
&= \sum_{B_1,B_2,\cdots,B_s} \sum_{i=1}^{s} \frac{p_{AB_1}p_{AB_2}\cdots p_{AB_s}}{f_A^{s-1} f_{B_i}} \beta_{B_i} \\
&= \sum_{i=1}^{s} \sum_{B_j (j=1,2,\cdots,s;j\neq i)} \frac{\prod_{j=1,2,\cdots,s;j\neq i} p_{AB_j}}{f_A^{s-1}} (\frac{p_{AB_i}}{f_{B_i}}\beta_{B_i} - \frac{p_{Ab_i}}{f_{b_i}}\beta_{B_i}) \\
&= \sum_{i=1}^{s} \frac{\prod_{j=1,2,\cdots,s;j\neq i}(p_{AB_j}+p_{Ab_j})}{f_A^{s-1}} (\frac{p_{AB_i}}{f_{B_i}}\beta_{B_i} - \frac{p_{Ab_i}}{f_{b_i}}\beta_{B_i}) \\
&= \sum_{i=1}^{s} (\frac{p_{AB_i}}{f_{B_i}}\beta_{B_i} - \frac{p_{Ab_i}}{f_{b_i}}\beta_{B_i}) \\
&= \sum_{i=1}^{s} (p_{A|B_i} - p_{A|b_i})\beta_{B_i} \\
&= \sum_{i=1}^{s} \frac{D_{AB_i}}{f_{B_i} f_{b_i}} \beta_{B_i}.
\end{aligned} \tag{26}
$$

Thus we have

$$
E(\boldsymbol{z}_A) = \sum_{B_j (j=1,2,\cdots,s;j\neq i)} r_{AB_j} \frac{\beta_{B_j}}{s_{B_j}} \tag{27}
$$

(d) For non-risk SNPs, their corresponding β are equal to zero. Hence, we have

$$
E(\boldsymbol{z}) = \boldsymbol{R}\boldsymbol{S}^{-1}\boldsymbol{\beta}. \tag{28}
$$

3. Combining covariance and expectation together, due to the central limit theorem, we have

$$
\boldsymbol{z} \sim N(\boldsymbol{R}\boldsymbol{S}^{-1}\boldsymbol{\beta}, \boldsymbol{R}) \tag{29}
$$

and

$$
\hat{\boldsymbol{f}}_{\boldsymbol{1}} - \hat{\boldsymbol{f}}_{\boldsymbol{0}} = \boldsymbol{S}^{-1}\boldsymbol{z} \sim N(\boldsymbol{S}\boldsymbol{R}\boldsymbol{S}^{-1}\boldsymbol{\beta}, \boldsymbol{S}\boldsymbol{R}\boldsymbol{S}). \tag{30}
$$

That ends the proof of Property 2. □

1.3 Connection between effect size distribution and liability-scale heritability

In PRStuning, we assume the β_m/s_m, the standardized effect of SNP m in terms of changing its allele frequencies between cases and controls, follows a point-normal mixture model:

$$\frac{\beta_m}{s_m} \overset{iid}{\sim} (1-\pi)\delta_0 + \pi N(0,\sigma^2), \tag{31}$$

where $s_m \approx \sqrt{2f_m(1-f_m)/N_e}$, f_m is the pooled allele frequencies, and N_e is the effective sample size defined by $N_e = \sqrt{\frac{4n_0n_1}{n_0+n_1}}$.

We consider the following linear model to derive the observed-scale heritability based on the distribution of the standardized effects:

$$Y = \alpha + \sum_{m=1}^{M} \tilde{\beta}_m \tilde{G}_m + e, \tag{32}$$

where Y is the affection status of the disease and $Y = 1$ (or 0) indicates the individual is from the case group (or control group). Here $\tilde{G}_m$ and G_m are the centered and raw genotype of SNP m, respectively. We use α and $\tilde{\beta}_m$ to represent the intercept and slope coefficient of $\tilde{G}_m$ in the linear model, respectively. As a residual, we have $E(e) = 0$. We further denote the types of the two alleles constituting the SNP as $\mathcal{A}_{m,1}$ and $\mathcal{A}_{m,2}$, i.e., $G_m = \mathcal{A}_{m,1} + \mathcal{A}_{m,2}$. With those notations, we have

$$Y = \alpha + \sum_{m=1}^{M} \tilde{\beta}_m[(\mathcal{A}_{m,1} - f_m) + (\mathcal{A}_{m,2} - f_m)] + e. \tag{33}$$

In Supplementary Equation (33), $\tilde{\beta}_m$ can be interpreted as the expected change of Y when $\mathcal{A}_{m,1}$ (or $\mathcal{A}_{m,2}$, we assume they have the same effect on the phenotype) increases one unit without influence from other SNPs (Agresti, 2012), i.e.,

$$\begin{aligned}
\tilde{\beta}_m &= E(Y|\mathcal{A}_{m,1}=1) - E(Y|\mathcal{A}_{m,1}=0) \\
&= P(Y=1|\mathcal{A}_{m,1}=1) - P(Y=1|\mathcal{A}_{m,1}=0) \\
&= \frac{P(\mathcal{A}_{m,1}=1|Y=1)P(Y=1)}{P(\mathcal{A}_{m,1}=1)} - \frac{P(\mathcal{A}_{m,1}=0|Y=1)P(Y=1)}{P(\mathcal{A}_{m,1}=0)} \\
&= \frac{[P(\mathcal{A}_{m,1}=1|Y=1) - P(\mathcal{A}_{m,1}=1|Y=0)]P(Y=1)P(Y=0)}{P(\mathcal{A}_{m,1}=1)P(\mathcal{A}_{m,1}=0)}.
\end{aligned} \tag{34}$$

The last equality holds due to $P(\mathcal{A}_{m,1}=1) = P(\mathcal{A}_{m,1}=1|Y=1)P(Y=1) + P(\mathcal{A}_{m,1}=1|Y=0)P(Y=0)$ and $P(\mathcal{A}_{m,1}=0) = P(\mathcal{A}_{m,1}=0|Y=1)P(Y=1) + P(\mathcal{A}_{m,1}=0|Y=0)P(Y=0)$.

By definitions, we have $\beta_m = P(\mathcal{A}_{m,1} = 1|Y = 1) - P(\mathcal{A}_{m,1} = 1|Y = 0)$ and $f_m = P(\mathcal{A}_{m,1} = 1)$. Let υ represent the proportion of cases among the individuals selected in the GWAS. With those notations, $\tilde{\beta}_m$ can be represented in the following form based on Supplementary Equation (34):

$$\tilde{\beta}_m = \frac{\beta\upsilon(1-\upsilon)}{f_m(1-f_m)}. \tag{35}$$

Therefore, the observed-scale heritability is

$$\begin{aligned} h_o^2 &= \frac{Var(\sum_{m=1}^{M} \tilde{\beta}_m \tilde{G}_m)}{Var(Y)} \\ &= \frac{2\sum_{m=1}^{M} E(\tilde{\beta}_m^2 f_m(1-f_m))}{\upsilon(1-\upsilon)} \\ &= 2\upsilon(1-\upsilon)\sum_{m=1}^{M} E\left(\frac{\beta_m^2}{f_m(1-f_m)}\right). \end{aligned} \tag{36}$$

With the prior distribution of the standardized effect Supplementary Equation (31), we have

$$E\left(\frac{\beta^2}{f_m(1-f_m)}\right) = \frac{2\pi\sigma^2}{N_e}. \tag{37}$$

Plugging in this equality into Supplementary Equation (36), the observed-scale heritability is

$$h_o^2 = \frac{4\upsilon(1-\upsilon)M\pi\sigma^2}{N_e}. \tag{38}$$

Note that the case proportion (υ) among the GWAS individuals is usually higher than the prevalence (κ) of the disease in the retrospective case-control design. The term $\upsilon(1-\upsilon)$ in the observed heritability is for ascertainment of the increased proportion of the cases. If the GWAS summary statistics are derived from biobank data, in which the individuals are selected not based on the affection status of the disease, we have $\upsilon = \kappa$.

Lee et al. derived the relationship between the observed-scale heritability (h_o^2) and the liability-scale heritability (h_l^2) with the following equality:

$$h_l^2 = h_o^2 \frac{\kappa(1-\kappa)}{\phi(\Phi^{-1}(\kappa))^2} \frac{\kappa(1-\kappa)}{\upsilon(1-\upsilon)}, \tag{39}$$

where ϕ and Φ are the probability density function and cumulative density function of the standard normal distribution $N(0,1)$, respectively. Combining this equality and Supplementary Equation (38), we have

$$h_l^2 = \frac{4M\pi\sigma^2}{N_e} \frac{\kappa^2(1-\kappa)^2}{\phi(\Phi^{-1}(\kappa))^2}. \tag{40}$$

1.4 Derivation for Gibbs sampling-based SAME algorithm to estimate AUC among genome-wide SNPs

Recall the joint distribution of z-scores:

$$\boldsymbol{z}|\boldsymbol{\beta} \sim N(\boldsymbol{R}\boldsymbol{S}^{-1}\boldsymbol{\beta}, \boldsymbol{R}), \tag{41}$$

where $\boldsymbol{S}$ is a diagonal matrix with diagonal elements encoding the standard errors of observed allele frequency differences, and $\boldsymbol{R}$ is the LD matrix representing the pairwise correlations of SNP genotypes.

We further assume each element of $\boldsymbol{\beta}$ follows a point-normal distribution:

$$\frac{\beta_m}{s_m} \sim (1-\pi)\delta_0 + \pi N(0, \sigma^2). \tag{42}$$

We can rewrite the above distribution by introducing dummy variables $\gamma_m \in \{0, 1\}$ $(m = 1, \ldots, M)$ indicating whether each SNP has an effect on the disease. The transformed form of the distribution is:

$$\frac{\beta_m}{s_m}|\gamma_m \sim \begin{cases} \delta_0 & \text{if } \gamma_m = 0 \\ N(0, \sigma^2) & \text{if } \gamma_m = 1 \end{cases} \tag{43}$$

and $\gamma_m \sim Bernoulli(\pi)$. We denote $\boldsymbol{\gamma} = (\gamma_1, \ldots, \gamma_M)$.

The log-likelihood of the complete data, including z-scores and augmented parameters $(\boldsymbol{\beta}, \boldsymbol{\gamma})$, is expressed

as follows:

$$
\begin{aligned}
& \log P(\boldsymbol{z}, \boldsymbol{\beta}, \boldsymbol{\gamma} \big| \pi, \sigma^2) \\
= \ & \log P(\boldsymbol{z}|\boldsymbol{\beta}) + \log P(\boldsymbol{\beta}|\boldsymbol{\gamma}, \sigma^2) + \log P(\boldsymbol{\gamma}|\pi) \\
= \ & -\frac{1}{2}(\boldsymbol{z} - \boldsymbol{R}\boldsymbol{S}^{\boldsymbol{-1}}\boldsymbol{\beta})^T \boldsymbol{R}^{-1}(\boldsymbol{z} - \boldsymbol{R}\boldsymbol{S}^{\boldsymbol{-1}}\boldsymbol{\beta}) + \sum_{m=1}^{M}(1-\gamma_m)\log\delta_0 + \frac{1}{2}\sum_{m=1}^{M}\gamma_m[\log(\sigma^{-2}) - \frac{\beta_m^2}{s_m^2}\sigma^{-2}] \\
& + \sum_{m=1}^{M}[(1-\gamma_m)\log(1-\pi) + (1-\gamma_m)\log\pi] + Const \\
= \ & -\frac{1}{2}\boldsymbol{\beta^T S^{-1} R S^{-1} \beta} + \boldsymbol{\beta^T S^{-1} z} + \sum_{m=1}^{M}(1-\gamma_m)\log\delta_0 + \frac{1}{2}\sum_{m=1}^{M}\gamma_m[\log(\sigma^{-2}) - \frac{\beta_m^2}{s_m^2}\sigma^{-2}] \\
& + \sum_{m=1}^{M}[(1-\gamma_m)\log(1-\pi) + (1-\gamma_m)\log\pi] + Const \\
= \ & -\frac{1}{2}\frac{\beta_m^2}{s_m^2} - \frac{\beta_m}{s_m}\sum_{m'\neq m}\frac{\beta_{m'}}{s_{m'}}R_{mm'} - \frac{1}{2}\sum_{m'\neq m}\frac{\beta_{m'}^2}{s_{m'}^2} - \frac{1}{2}\sum_{m'\neq m}\sum_{m''\neq m'}\frac{\beta_{m'}\beta_{m''}}{s_{m'}s_{m''}}R_{m'm''} \\
& + \frac{\beta_m z_m}{s_m} + \sum_{m'\neq m}\frac{\beta_{m'}z_{m'}}{s_{m'}} + \sum_{m=1}^{M}(1-\gamma_m)\log\delta_0 + \frac{1}{2}\sum_{m=1}^{M}\gamma_m[\log(\sigma^{-2}) - \frac{\beta_m^2}{s_m^2}\sigma^{-2}] \\
& + \sum_{m=1}^{M}[(1-\gamma_m)\log(1-\pi) + (1-\gamma_m)\log\pi] + Const,
\end{aligned}
\tag{44}
$$

where s_m is the m-th diagonal elements of $\boldsymbol{S}$ and *const* denotes a constant unrelated with any parameter.

Based on this log-likelihood, we can derive the conditional distribution of β_m given all other parameters:

$$
\beta_m|\boldsymbol{z}, \boldsymbol{\beta}_{-m}, \gamma_m, \sigma^2 \sim \begin{cases} \delta_0 & \text{if } \gamma_m = 0 \\ N(s_m\mu_m, \lambda s_m^2), & \text{if } \gamma_m = 1 \end{cases}, \tag{45}
$$

where $\lambda^{-1} = 1 + \sigma^{-2}$ and $\mu_m = \lambda(z_m - \sum_{m'\neq m}\frac{R_{mm'}\beta_{m'}}{s_{m'}})$. In the above formula, we denote $\boldsymbol{\beta}_{-m}$ as the vector of effect sizes of all SNPs except SNP m.

It is not trivial to deduct the conditional distribution of γ_m given the values of the other parameters since the probability density function of δ_0 is actually inaccessible. To tackle this challenge, we integrate the Dirac delta function over β_m, and calculate the following probabilities:

$$
\begin{aligned}
P(\boldsymbol{z}|\gamma_m = 0, \boldsymbol{\beta}_{-m}) = & P(\boldsymbol{z}|\beta_m = 0, \boldsymbol{\beta}_{-m}) \\
& \propto \exp\left[-\frac{1}{2}\sum_{m'\neq m}\frac{\beta_{m'}^2}{s_{m'}^2} - \frac{1}{2}\sum_{m'\neq j}\sum_{m''\neq j'}\frac{\beta_{m'}\beta_{m''}}{s_{m'}s_{m''}}R_{m'm''} + \sum_{m'\neq m}\frac{\beta_{m'}z_{m'}}{s_{m'}}\right] \\
& \equiv C
\end{aligned}
\tag{46}
$$

and

$$\begin{aligned}
P(\boldsymbol{z}|\gamma_m=1,\boldsymbol{\beta}_{-m},\sigma^2)=&\int P(\boldsymbol{z}|\beta_m,\boldsymbol{\beta}_{-m})P(\beta_m|\gamma_m=1,\sigma^2)d\beta_m\\
\propto&\int\exp\left[-\frac{1}{2}\sum_{m'\neq m}\frac{\beta_{m'}^2}{s_{m'}^2}-\frac{1}{2}\sum_{m'\neq m}\sum_{m''\neq m'}\frac{\beta_{m'}\beta_{m''}}{s_{m'}s_{m''}}R_{m'm''}+\sum_{m'\neq m}\frac{\beta_{m'}z_{m'}}{s_{m'}}\right.\\
&\left.-\frac{1}{2}\frac{\beta_m^2}{s_m^2}-\frac{\beta_m}{s_m}\sum_{m'\neq m}\frac{\beta_{m'}}{s_{m'}}R_{mm'}+\frac{\beta_m z_m}{s_m}\right]\frac{1}{\sqrt{2\pi\sigma^2}}\exp\left[-\frac{1}{2}\frac{\beta_m^2}{s_m^2\sigma^2}\right]d\beta_m\\
=&C\frac{1}{\sqrt{2\pi\sigma^2}}\int\exp\left[-\frac{1}{2}\frac{\beta_m^2}{s_m^2}-\frac{\beta_m}{s_m}\sum_{m'\neq m}\frac{\beta_{m'}}{s_{m'}}R_{mm'}+\frac{\beta_m z_m}{s_m}-\frac{1}{2}\frac{\beta_m^2}{s_m^2\sigma^2}\right]d\beta_m\\
=&C\sqrt{\frac{1}{1+\sigma^2}}\exp\left[\frac{(\frac{z_m}{s_m}-\frac{\beta_m}{s_m}\sum_{m'\neq m}\frac{\beta_{m'}}{s_{m'}}R_{mm'})^2}{2(1+\sigma^{-2})}\right]\\
=&C\sqrt{\frac{\lambda}{\sigma^2}}\exp\left(\frac{\mu_m^2}{2\lambda}\right).
\end{aligned}\tag{47}$$

Hence, the conditional probability of γ_m is:

$$\begin{aligned}
h_m\equiv&P(\gamma_m=1|\boldsymbol{z},\boldsymbol{\beta}_{-m},\pi,\sigma^2)\\
=&\frac{P(\gamma_m=1)P(\boldsymbol{z}|\gamma_m=1,\boldsymbol{\beta}_{-j},\sigma^2)}{P(\gamma_m=0)P(\boldsymbol{z}|\gamma_m=0,\boldsymbol{\beta}_{-m})+P(\gamma_m=1)P(\boldsymbol{z}|\gamma_m=1,\boldsymbol{\beta}_{-m},\sigma^2)}\\
=&\frac{\pi\sqrt{\frac{\lambda}{\sigma^2}}\exp(\frac{\mu_m^2}{2\lambda})}{(1-\pi)+\pi\sqrt{\frac{\lambda}{\sigma^2}}\exp(\frac{\mu_m^2}{2\lambda})}.
\end{aligned}\tag{48}$$

With this probability, we have

$$\gamma_m|\boldsymbol{z},\boldsymbol{\beta}_{-m},\pi,\sigma^2\sim Bernoulli(h_m).\tag{49}$$

Back to the log likelihood of the augmented data presented in Supplementary Equation (44), we can also derive the conditional distribution of π and σ^{-2} given other related parameters as follows:

$$\pi|\boldsymbol{\gamma}\sim Beta\left(\sum_{m=1}^{M}\gamma_m+1,M-\sum_{m=1}^{M}\gamma_m+1\right);\tag{50}$$

$$\sigma^{-2}|\boldsymbol{\beta},\boldsymbol{\gamma}\sim Gamma\left(\frac{1}{2}\sum_{m=1}^{M}\gamma_m+1,\frac{1}{2}\sum_{m=1}^{M}\beta_m^2\gamma_m\right).\tag{51}$$

In summary, Supplementary Equations (45), (49), (50) and (51) together provide us steps of a standard Gibbs sampler for sampling parameters $\boldsymbol{\beta}$, $\boldsymbol{\gamma}$, π and σ^2 with their joint probability proportional to $P(\boldsymbol{z},\boldsymbol{\beta},\boldsymbol{\gamma}|\pi,\sigma^2)$.

For the SAME algorithm (Doucet et al., 2002), we need to construct a Gibbs sampler for sampling $\{\pi,\sigma^2\}$ and D artificial replications of nuisance parameters $\boldsymbol{\beta}(1),\boldsymbol{\gamma}(1),\ldots,\boldsymbol{\beta}(D),\boldsymbol{\gamma}(D)$ simultaneously with

their joint probability proportional to

$$q_D\big(\pi, \sigma^2, \{\boldsymbol{\beta}(d), \boldsymbol{\gamma}(d)\}_{d=1}^D \big| \boldsymbol{z}\big) \propto \prod_{d=1}^D P\big(\boldsymbol{z}, \boldsymbol{\beta}(d), \boldsymbol{\gamma}(d) \big| \pi, \sigma^2\big). \tag{52}$$

By making simple changes to the above derived standard Gibbs sampler, we can get a new Gibbs sampler with the joint property Supplementary Equation (52).

First, as the replications of $\{\boldsymbol{\beta}, \boldsymbol{\gamma}\}$ are statistically independent conditional upon $\{\pi, \sigma^2\}$, i.e,

$$q_D\big(\{\boldsymbol{\beta}(d), \boldsymbol{\gamma}(d)\}_{d=1}^D \big| \boldsymbol{z}, \pi, \sigma^2\big) = \prod_{d=1}^D p(\boldsymbol{\beta}(d), \boldsymbol{\gamma}(d) \big| \boldsymbol{z}, \pi, \sigma^2), \tag{53}$$

we can still use the identical forms of Supplementary Equations (45) and (49) to sample them.

For $\{\pi, \sigma^2\}$, their conditional density satisfies

$$q_D\big(\pi \big| \boldsymbol{z}, \{\boldsymbol{\beta}(d), \boldsymbol{\gamma}(d)\}_{d=1}^D, \sigma^2\big) = \prod_{d=1}^D p(\pi \big| \boldsymbol{z}, \boldsymbol{\beta}(d), \boldsymbol{\gamma}(d), \sigma^2); \tag{54}$$

$$q_D\big(\sigma^2 \big| \boldsymbol{z}, \{\boldsymbol{\beta}(d), \boldsymbol{\gamma}(d)\}_{d=1}^D, \pi\big) = \prod_{d=1}^D p(\sigma^2 \big| \boldsymbol{z}, \boldsymbol{\beta}(d), \boldsymbol{\gamma}(d), \pi). \tag{55}$$

Since both $p(\pi \big| \boldsymbol{z}, \boldsymbol{\beta}(d), \boldsymbol{\gamma}(d), \sigma^2)$ and $p(\sigma^2 \big| \boldsymbol{z}, \boldsymbol{\beta}(d), \boldsymbol{\gamma}(d), \pi)$ are members of exponential family according to Supplementary Equations (50), (51), sampling from $q_D\big(\pi \big| \boldsymbol{z}, \{\boldsymbol{\beta}(d), \boldsymbol{\gamma}(d)\}_{d=1}^D, \sigma^2\big)$ and $q_D\big(\sigma^2 \big| \boldsymbol{z}, \{\boldsymbol{\beta}(d), \boldsymbol{\gamma}(d)\}_{d=1}^D, \pi\big)$ are straightforward as the product of conditional densities given each replicate are also members of exponential family. We have

$$\pi \big| \{\boldsymbol{\gamma}(d)\}_{d=1}^D \sim Beta\left(\sum_{d=1}^D \sum_{m=1}^M \gamma_m(d) + D, MD - \sum_{d=1}^D \sum_{m=1}^M \gamma_m(d) + D\right) \tag{56}$$

and

$$\sigma^{-2} \big| \{\boldsymbol{\beta}(d), \boldsymbol{\gamma}(d)\}_{d=1}^D \sim Gamma\left(\frac{1}{2} \sum_{d=1}^D \sum_{m=1}^M \gamma_m(d) + D, \frac{1}{2} \sum_{d=1}^D \sum_{m=1}^M \beta_j(d)^2 \gamma_m(d)\right). \tag{57}$$

1.5 Rationale of alleviating overfitting with a Bayes estimator

In this section, we first provide a theoretical demonstration of how overfitting happens if we simply plug the summary statistics from the training data into the derived AUC function below:

$$\text{AUC} = \Phi(\Delta), \tag{58}$$

where

$$\Delta := \frac{2 \sum_{m=1}^M \omega_m \delta_m}{\sqrt{\tau_0^2 + \tau_1^2}} \text{ and } \tau_j^2 = \sum_{m=1}^M \omega_m^2 s_{j,m}^2 + 2 \sum_{m_1 < m_2} \omega_{m_1} \omega_{m_2} R_{m_1,m_2} s_{j,m_1} s_{j,m_2}. \tag{59}$$

From the equations, we know that AUC is monotonically increasing with respect to Δ, and we have $\Delta \propto \zeta := \sum_{m=1}^{M} \omega_m \delta_m$, which is directly influenced by the SNP effects on the disease. The distribution of z_m is $N(\delta_m/s_m, 1)$. To demonstrate the overfitting of AUC, we compare the expectation of $\hat{\zeta}_{train} := \sum_{m=1}^{M} \omega_m s_m z_m$, in which δ_m in ζ is replaced by its observed value $s_m z_m$ from training data, with the expectation of ζ.

We consider a simplified scenario in which pre-selected SNPs are independent and P+T is used to derive the SNP weights, i.e.,

$$\omega_m = \begin{cases} z_m s_m, & \text{if } |z_m| \geq t \\ 0, & \text{otherwise} \end{cases}, \tag{60}$$

where t is the threshold used to filter SNPs based on z-scores to construct PRS.

In this scenario, the expectation of ζ is

$$\begin{aligned} E(\zeta) &= \sum_{m=1}^{M} s_m \delta_m P(|z_m| \geq t) E(z_m \| z_m| \geq t) \\ &= \sum_{m=1}^{M} s_m \delta_m P(|z_m| \geq t) \frac{\int_{-\infty}^{-t} z_m \phi(z_m - \delta_m/s_m) dz_m + \int_{t}^{\infty} z_m \phi(z_m - \delta_m/s_m) dz_m}{P(|z_m| \geq t)} \\ &= \sum_{m=1}^{M} s_m \delta_m \left(\int_{-\infty}^{-t-\delta_m/s_m} u_m \phi(u_m) du_m + \frac{\delta_m}{s_m} \int_{-\infty}^{-t-\delta_m/s_m} \phi(u_m) du_m \right. \\ &\quad \left. + \int_{t-\delta_m/s_m}^{\infty} u_m \phi(u_m) du_m + \frac{\delta_m}{s_m} \int_{t-\delta_m/s_m}^{\infty} \phi(u_m) du_m \right) \\ &= \sum_{m=1}^{M} \left(\delta_m^2 P(|z_m| \geq t) + s_m \delta_m (\phi(t - \delta_m/s_m) - \phi(-t - \delta_m/s_m)) \right) \end{aligned} \tag{61}$$

where $u_m := z_m - \delta_m/s_m \sim N(0,1)$, $P(|z_m| \geq t) = 1 - \Phi(t - \delta_m/s_m) + \Phi(-t - \delta_m/s_m)$, and ϕ and Φ are probability density function and cumulative density function of the standard normal distribution $N(0,1)$, respectively. Since $\phi(t - \delta_m/s_m) - \phi(-t - \delta_m/s_m)$ has the same sign with δ_m, we have

$$s_m \delta_m (\phi(t - \delta_m/s_m) - \phi(-t - \delta_m/s_m)) > 0, \tag{62}$$

which represents the influence on AUC of selecting only SNPs with large effect sizes.

In contrast, the expectation of $\hat{\zeta}$ is

$$\begin{aligned}
E(\hat{\zeta}_{train}) &= \sum_{m=1}^{M} s_m^2 P(|z_m| \geq t) E(z_m^2 || z_m| \geq t) \\
&= \sum_{m=1}^{M} s_m^2 P(|z_m| \geq t) \frac{\int_{-\infty}^{-t} z_m^2 \phi(z_m - \delta_m/s_m) dz_m + \int_t^{\infty} z_m^2 \phi(z_m - \delta_m/s_m) dz_m}{P(|z_m| \geq t)} \\
&= \sum_{m=1}^{M} s_m^2 \left(\int_{-\infty}^{-t-\delta_m/s_m} u_m^2 \phi(u_m) du_m + 2\frac{\delta_m}{s_m} \int_{-\infty}^{-t-\delta_m/s_m} u_m \phi(u_m) du_m \right. \\
&\quad + \frac{\delta_m^2}{s_m^2} \int_{-\infty}^{-t-\delta_m/s_m} \phi(u_m) du_m + \int_{t-\delta_m/s_m}^{\infty} u_m^2 \phi(u_m) du_m \\
&\quad \left. + 2\frac{\delta_m}{s_m} \int_{t-\delta_m/s_m}^{\infty} u_m \phi(u_m) du_m + \frac{\delta_m^2}{s_m^2} \int_{t-\delta_m/s_m}^{\infty} \phi(u_m) du_m \right) \\
&= \sum_{m=1}^{M} s_m^2 \left(\Phi(-t - \delta_m/s_m) - (-t - \frac{\delta_m}{s_m}) \phi(-t - \delta_m/s_m) - 2\frac{\delta_m}{s_m} \phi(-t - \delta_m/s_m) \right. \\
&\quad + \frac{\delta_m^2}{s_m^2} \Phi(-t - \delta_m/s_m) + 1 - \Phi(t - \delta_m/s_m) + (t - \frac{\delta_m}{s_m}) \phi(t - \delta_m/s_m) \\
&\quad \left. + 2\frac{\delta_m}{s_m} \phi(t - \delta_m/s_m) + \frac{\delta_m^2}{s_m^2} (1 - \Phi(t - \delta_m/s_m)) \right) \\
&= \sum_{m=1}^{M} \left(\delta_m^2 P(|z_m| \geq t) + s_m \delta_m (\phi(t - \delta_m/s_m) - \phi(-t - \delta_m/s_m)) \right. \\
&\quad \left. + t s_m^2 (\phi(t - \delta_m/s_m) + \phi(-t - \delta_m/s_m)) + s_m^2 P(|z_m| \geq t) \right) \qquad (63)
\end{aligned}$$

Therefore, we have

$$E(\hat{\zeta}_{train}) - E(\zeta) = \sum_{m=1}^{M} (t s_m^2 (\phi(t - \delta_m/s_m) + \phi(-t - \delta_m/s_m)) + s_m^2 P(|z_m| \geq t)) \geq 0. \qquad (64)$$

That inequality explains why overfitting happens if we directly plug the summary statistics from the training data into the derived AUC function. We further define

$$\begin{aligned}
g_m(t) &= t(\phi(t - \delta_m/s_m) + \phi(-t - \delta_m/s_m)) + P(|z_m| \geq t) \\
&= t(\phi(t - \delta_m/s_m) + \phi(-t - \delta_m/s_m)) + (1 - \Phi(t - \delta_m/s_m) + \Phi(-t - \delta_m/s_m)) \qquad (65)
\end{aligned}$$

Taking derivative of $g_m(t)$, we get

$$g'_m(t) = t(\phi'(t - \delta_m/s_m) - \phi'(-t - \delta_m/s_m)) < 0, \qquad (66)$$

which indicates the overfitting phenomenon will be more severe when more SNPs are involved in PRS calculation. Especially, if all pre-selected SNPs are used to construct PRS without filtering ($t = 0$), we have

$$E(\hat{\zeta}_{train}) - E(\zeta) = \sum_{m=1}^{M} s_m^2. \qquad (67)$$

Recalling $\Delta \propto \sum_{m=1}^{M} \omega_m \delta_m$, here we consider how to estimate δ_m in terms of minimizing the Bayes risk $R(\hat{\delta}_m)$, where $\hat{\delta}_m$ is an estimator of δ_m. The Bayes risk reads

$$\begin{aligned} R(\hat{\delta}_m) &= E_{\delta_m}\left((\hat{\delta}_m - \delta_m)^2 \,\middle|\, |z_m| \geq t\right) \\ &= \int_{-\infty}^{\infty}\left(\int_{|z_m|\geq t}(\hat{\delta}_m - \delta_m)^2 \frac{p(z_m|\delta_m)}{P(|z_m| \geq t)} dz_m\right) p(\delta_m||z_m| \geq t) d\delta_m \\ &= \frac{1}{P(|z_m| \geq t)} \int_{-\infty}^{\infty}\left(\int_{|z_m|\geq t}(\hat{\delta}_m - \delta_m)^2 p(z_m|\delta_m) dz_m\right) p(\delta_m) d\delta_m \\ &= \frac{1}{P(|z_m| \geq t)} \int_{|z_m|\geq t}\left(\int_{-\infty}^{\infty}(\hat{\delta}_m - \delta_m)^2 p(\delta_m|z_m) d\delta_m\right) p(z_m) dz_m. \end{aligned} \quad (68)$$

The last equality holds by Fubini's theorem. From the last equality, we can conclude that the posterior expectation of δ_m, namely the Bayes estimator $E(\delta_m|z_m)$, is the minimizer of the Bayes risk even if SNPs for constructing PRS are selected based on z-scores/p-values. With the improved estimation accuracy, we plug the Bayes estimator of δ_m into the AUC function in PRStuning to alleviate the overfitting.

1.6 Quality control procedure for UK Biobank data

We used the following quality control criteria to select SNPs and individuals in the UK Biobank data:

- Minor allele frequency control: The SNPs with minor allele frequencies $\geq 5\%$ were selected;
- Genotype-missing rate control for SNPs: The SNPs with genotype-missing rates $\leq 5\%$ were preserved;
- Hardy-Weinberg equilibrium (HWE) control: The SNPs with p-values$\geq 1 \times 10^{-4}$ in the HWE tests were included;
- Imputation quality control: The SNPs with imputed information score INFO > 0.8 were included;
- Genotype-missing rate control for individuals: The individuals with genotype-missing rate $\leq 1\%$ were included;
- Population stratification control: The selected individuals reported themselves as white British ancestry.
- Relatedness control: The selected individuals have no family relationships among them.

1.7 Extracting phenotypes from UK Biobank

For three diseases, we extracted cases based on ICD-9 codes (FIELD ID: 41271), ICD-10 codes (FIELD ID: 41270), OPCS-4 codes (FIELD ID: 41272), self-reported questionnaire (FIELD ID: 20002) or operation code (FIELD ID: 20004):

- For coronary artery diseases, we selected cases with ICD-9 codes of 410-412; ICD-10 codes of I21-I23, I241 and I252; OPCS-4 codes of K40.1-40.4, K41.1-41.4, K45.1-45.5, K49.1-49.2, K49.8-49.9, K50.2, K75.1-75.4, K75.8-75.9; self-reported questionnaire of code 1075; or operation codes of 1070 and 1095.
- For inflammatory bowel disease, we selected cases with ICD-9 code of 555; ICD-10 code of K50; or self-reported history of Crohn's disease, ulcerative colitis, and inflammatory bowel disease.
- For type 2 diabetes, we selected cases with ICD-9 code of K51; ICD-10 code of E11; or self-reported history of type 2 diabetes (1223).

Supplementary Tables

Method	T2D	CAD	IBD	BC
P+T (p-value <= 1)	0.521 (0.519)	0.510 (0.515)	0.526 (0.518)	0.523 (0.514)
P+T (p-value <= 5e−1)	0.521 (0.520)	0.510 (0.515)	0.526 (0.519)	0.523 (0.515)
P+T (p-value <= 1e−1)	0.523 (0.523)	0.511 (0.513)	0.530 (0.520)	0.523 (0.515)
P+T (p-value <= 5e−2)	0.524 (0.523)	0.511 (0.515)	0.532 (0.526)	0.523 (0.517)
P+T (p-value <= 1e−2)	0.523 (0.524)	0.510 (0.512)	0.535 (0.524)	0.521 (0.515)
P+T (p-value <= 5e−3)	0.522 (0.525)	0.510 (0.511)	0.534 (0.523)	0.520 (0.514)
P+T (p-value <= 1e−3)	0.523 (0.523)	0.508 (0.506)	0.533 (0.520)	0.516 (0.512)
P+T (p-value <= 5e−4)	0.523 (0.522)	0.508 (0.506	0.532 (0.520)	0.515 (0.506)
P+T (p-value <= 1e−4)	0.523 (0.521)	0.504 (0.506)	0.531 (0.520)	0.513 (0.506)
P+T (p-value <= 5e−5)	0.521 (0.520)	0.504 (0.506)	0.530 (0.526)	0.513 (0.507)
C+T (p-value <= 1)	0.545 (0.554)	0.521 (0.545)	0.531 (0.559)	0.514 (0.521)
C+T (p-value <= 5e−1)	0.545 (0.555)	0.521 (0.545)	0.531 (0.559)	0.515 (0.521)
C+T (p-value <= 5e−2)	0.551 (0.552)	0.524 (0.545)	0.536 (0.563)	0.516 (0.522)
C+T (p-value <= 5e−3)	0.567 (0.552)	0.532 (0.543)	0.547 (0.569)	0.523 (0.526)
C+T (p-value <= 5e−4)	0.583 (0.553)	0.541 (0.542)	0.563 (0.578)	0.535 (0.529)
C+T (p-value <= 5e−5)	0.581 (0.562)	0.547 (0.540)	0.569 (0.581)	0.548 (0.540)
C+T (p-value <= 5e−6)	0.569 (0.563)	0.538 (0.537)	0.568 (0.573)	0.549 (0.543)
C+T (p-value <= 5e−7)	0.554 (0.550)	0.530 (0.528)	0.564 (0.569)	0.544 (0.544)
C+T (p-value <= 5e−8)	0.550 (0.550)	0.526 (0.526)	0.562 (0.567)	0.539 (0.539)
LDpred ($\pi = 1$)	0.581 (0.588)	0.530 (0.581)	0.568 (0.610)	0.530 (0.555)
LDpred ($\pi = 3e{-1}$)	0.586 (0.592)	0.531 (0.582)	0.574 (0.616)	0.533 (0.558)
LDpred ($\pi = 1e{-1}$)	0.594 (0.598)	0.535 (0.586)	0.586 (0.627)	0.540 (0.564)
LDpred ($\pi = 3e{-2}$)	0.610 (0.606)	0.543 (0.592)	0.602 (0.643)	0.552 (0.572)
LDpred ($\pi = 1e{-2}$)	0.535 (0.542)	0.554 (0.597)	0.583 (0.619)	0.566 (0.580)

LDpred ($\pi = 3e{-3}$)	0.514 (0.515)	0.528 (0.567)	0.518 (0.524)	0.579 (0.586)
LDpred ($\pi = 1e{-3}$)	0.510 (0.509)	0.508 (0.513)	0.508 (0.510)	0.587 (0.588)
LDpred ($\pi = 3e{-4}$)	0.619 (0.602)	0.505 (0.503)	0.502 (0.508)	0.590 (0.587)
LDpred ($\pi = 1e{-4}$)	0.602 (0.595)	0.502 (0.502)	0.504 (0.503)	0.587 (0.583)
LDpred ($\pi = 3e{-5}$)	0.589 (0.587)	0.500 (0.501)	0.500 (0.511)	0.583 (0.581)
LDpred ($\pi = 1e{-5}$)	0.581 (0.581)	0.500 (0.501)	0.607 (0.629)	0.576 (0.573)
LDpred2 ($\pi = 1$)	0.589 (0.588)	0.536 (0.583)	0.566 (0.607)	0.532 (0.563)
LDpred2 ($\pi = 3e{-1}$)	0.594 (0.592)	0.537 (0.585)	0.571 (0.612)	0.534 (0.565)
LDpred2 ($\pi = 1e{-1}$)	0.603 (0.598)	0.540 (0.588)	0.582 (0.623)	0.540 (0.570)
LDpred2 ($\pi = 3e{-2}$)	0.618 (0.607)	0.548 (0.595)	0.603 (0.642)	0.558 (0.584)
LDpred2 ($\pi = 1e{-2}$)	0.617 (0.601)	0.561 (0.602)	0.618 (0.657)	0.578 (0.598)
LDpred2 ($\pi = 3e{-3}$)	0.614 (0.609)	0.574 (0.600)	0.627 (0.660)	0.594 (0.604)
LDpred2 ($\pi = 1e{-3}$)	0.588 (0.569)	0.553 (0.579)	0.627 (0.658)	0.602 (0.603)
LDpred2 ($\pi = 3e{-4}$)	0.605 (0.588)	0.565 (0.580)	0.624 (0.651)	0.604 (0.600)
LDpred2 ($\pi = 1e{-4}$)	0.603 (0.593)	0.560 (0.565)	0.618 (0.641)	0.603 (0.596)
LDpred2 ($\pi = 3e{-5}$)	0.589 (0.585)	0.520 (0.520)	0.611 (0.633)	0.595 (0.590)
LDpred2 ($\pi = 1e{-5}$)	0.581 (0.578)	0.505 (0.509)	0.606 (0.625)	0.590 (0.585)

Supplementary Table 2: The predicted AUC by PRStuning and the actual AUC on testing data with PRS models built from P+T, C+T, LDpred, and LDpred2 using different parameters. The parameters for P+T and C+T are p-value thresholds for the two-sided Z test statistics in the GWAS summary data. The actual AUC values on testing data are summarized in parenthesis.

Disease	P+T	C+T	LDpred	LDpred2
T2D	66.5%	60.2%	58.5%	62.9%
CAD	58.6%	51.8%	68.1%	61.4%
IBD	65.2%	58.3%	62.9%	63.6%
BC	65.1%	52.4%	59.8%	66.4%

Supplementary Table 3: Sensitivity of the best-performing parameter chosen by PRStuning for four PRS methods on four diseases. Sensitivity is calculated using the cutoff selected by Youden's J statistic with the PRS model tuned by PRStuning.

Disease	P+T	C+T	LDpred	LDpred2
T2D	-0.663 (1.1%)	0.040 (1.5%)	0.231 (24.8%)	0.031 (24.4%)
CAD	-0.543 (6.2%)	0.895 (2.3%)	0.506 (24.9%)	0.115 (23.9%)
IBD	0.374 (0.6%)	0.845 (0.5%)	0.459 (16.7%)	-0.632 (13.7%)
BC	-0.587 (2.3%)	0.979 (4.8%)	-0.757 (26.4%)	-0.718 (23.0%)

Supplementary Table 4: Summary of ρ_{AUC} and rd_{AUC} when using PUMAS to predict AUCs for four PRS methods on four diseases. The rd_{AUC} are summarized in parenthesis.

Disease	PRStuning	P+T	C+T	LDpred	LDpred2
T2D	1h	3s	1.4min	7h	10min
CAD	1.3h	3s	1.9min	7h	11min
IBD	1.6h	3s	2.7min	8h	17min
BC	2h	3s	3.4min	8h	16min

Supplementary Table 5: Summary of running time for PRStuning and different PRS construction methods on real data. The running time is evaluated on an Intel Xeon E5-2680 CPU.

Method	CAD	IBD	T2D	BC
P+T (p-value <= 1)	1,968	3,710	1,654	4,783
P+T (p-value <= 5e−1)	1,024	1,967	1,060	2,456
P+T (p-value <= 1e−1)	228	500	222	569
P+T (p-value <= 5e−2)	120	313	116	297
P+T (p-value <= 1e−2)	30	93	32	78
P+T (p-value <= 5e−3)	21	54	17	41
P+T (p-value <= 1e−3)	6	22	6	11
P+T (p-value <= 5e−4)	5	19	5	7
P+T (p-value <= 1e−4)	1	16	5	5
P+T (p-value <= 5e−5)	1	13	3	4
C+T (p-value <= 1)	26,859	28,247	27,132	41,839
C+T (p-value <= 5e−1)	22,879	23,955	23,605	33,890
C+T (p-value <= 5e−2)	9,279	9,807	9,297	12,360
C+T (p-value <= 5e−3)	2,606	2,954	2,348	3,053
C+T (p-value <= 5e−4)	591	771	466	585
C+T (p-value <= 5e−5)	152	271	112	113
C+T (p-value <= 5e−6)	44	136	44	35
C+T (p-value <= 5e−7)	24	88	22	26
C+T (p-value <= 5e−8)	16	67	14	16

Supplementary Table 6: The number of SNPs selected by P+T and C+T with different p-value thresholds. The parameters for P+T and C+T are p-value thresholds for the two-sided Z test statistics in the GWAS summary data. Different from LDPred utilizing SNPs across the genome to build PRS, P+T and C+T select sets of independent SNPs and build PRS based on these sets. Please note that the selection step in C+T is related to the association strengths of SNPs, resulting in the inflation of observed effects among selected SNPs. Therefore, for C+T, we used genome-wide SNPs in PRStuning to address the effect inflation issue with Empirical Bayes theory.

Supplementary Figures

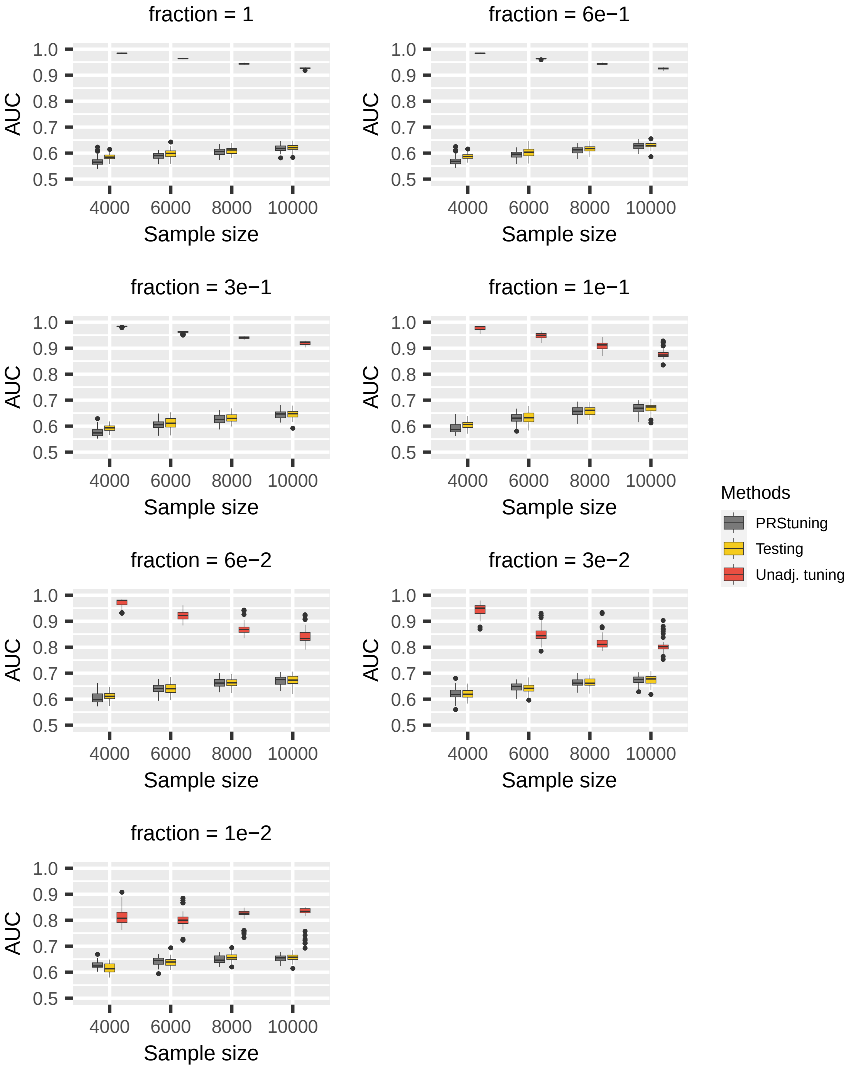

Supplementary Figure 1: AUC boxplots for LDpred2 in simulation experiments with correlated SNPs. Each box represents 50 replications and is presented as median values and the first and third quartiles. The upper/lower whisker extends from the hinge to the largest/smallest value at most 1.5 IQR from the hinge. We changed the proportion of risk SNPs from $\{1, 6e{-}1, 3e{-}1, 1e{-}1, 6e{-}2, 3e{-}2, 1e{-}2\}$ and the sample sizes of training data from $4,000$ to $10,000$. The grey, yellow, and red panels represent AUC predicted from PRStuning, AUC calculated from testing data, and the unadjusted AUC, respectively. The mean values of ρ_{AUC} and rd_{AUC} for each sample size are respectively $0.881, 0.975, 0.984, 0.979$ and $2.5\%, 1.9\%, 1.5\%, 1.5\%$.

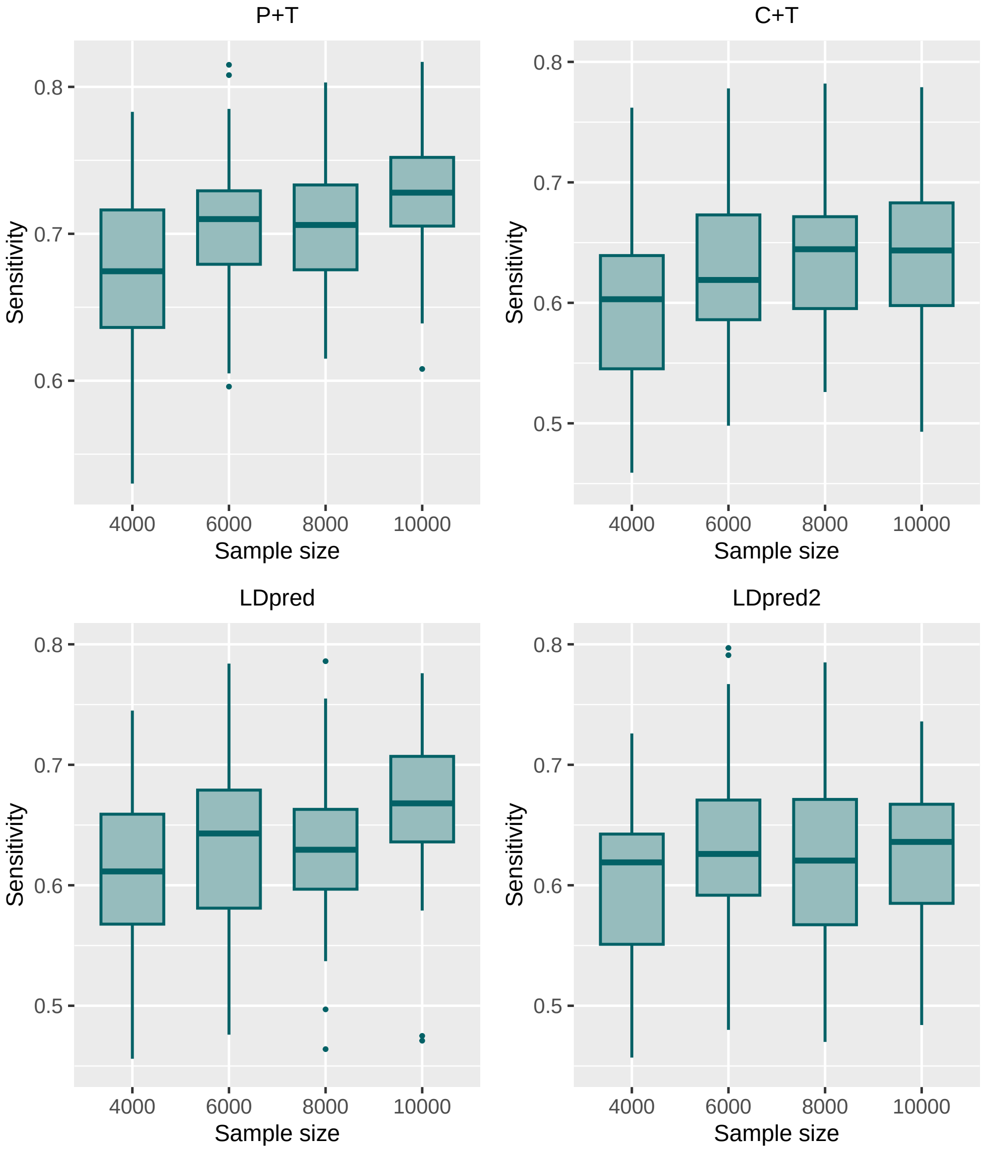

Supplementary Figure 2: Boxplots of sensitivity values for simulation experiments with independent SNPs (P+T) and correlated SNPs (C+T, LDpred, LDpred2). Each box represents 50 replications and is presented as median values and the first and third quartiles. The upper/lower whisker extends from the hinge to the largest/smallest value at most 1.5 IQR from the hinge. Sensitivity is calculated using the cutoff selected by Youden's J statistic with the PRS model tuned by PRStuning.

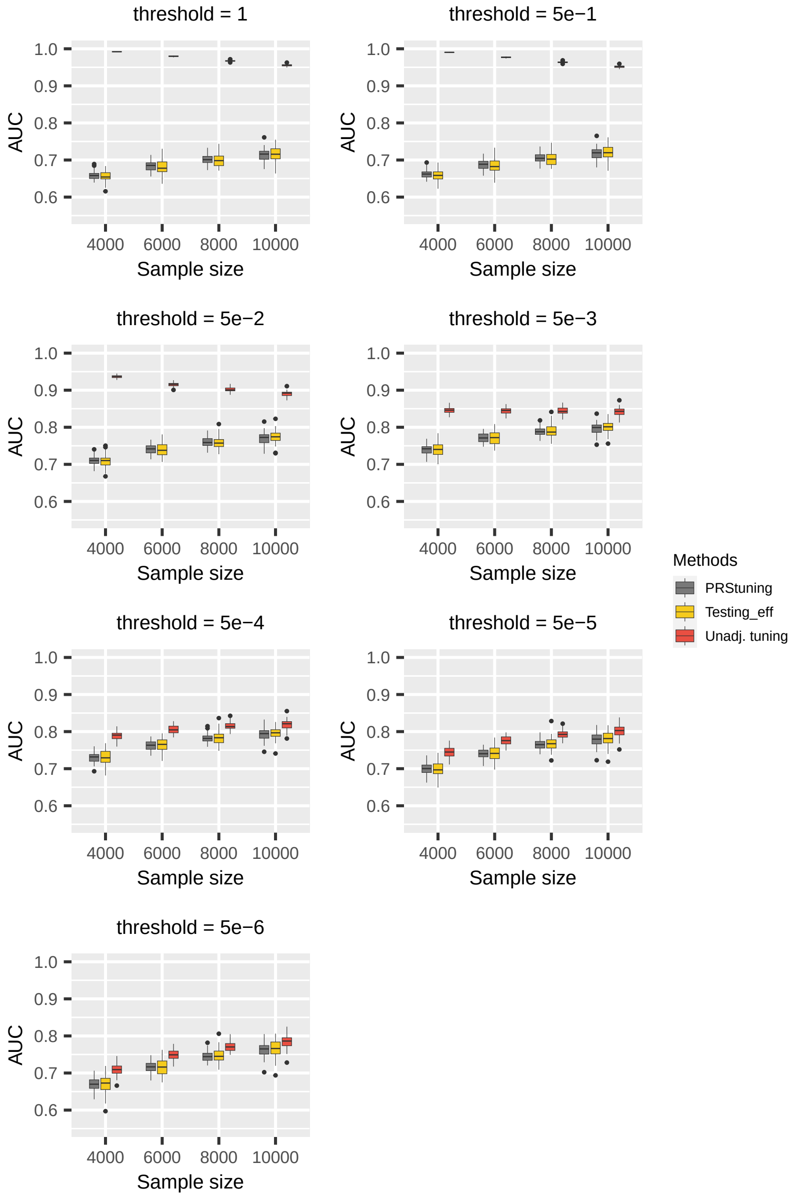

Supplementary Figure 3: AUC boxplots for P+T in the simulation experiments with independent SNPs. The MAF values of the training and testing data are heterogeneous. Each box represents 50 replications and is presented as median values and the first and third quartiles. The upper/lower whisker extends from the hinge to the largest/smallest value at most 1.5 IQR from the hinge. The grey, yellow, and red panels represent AUC predicted from PRStuning, AUC calculated from testing data, and the unadjusted AUC, respectively. The mean values of ρ_{AUC} and rd_{AUC} for each sample size are respectively $0.954, 0.976, 0.989, 0.991$ and $1.7\%, 1.1\%, 1.4\%, 1.1\%$.

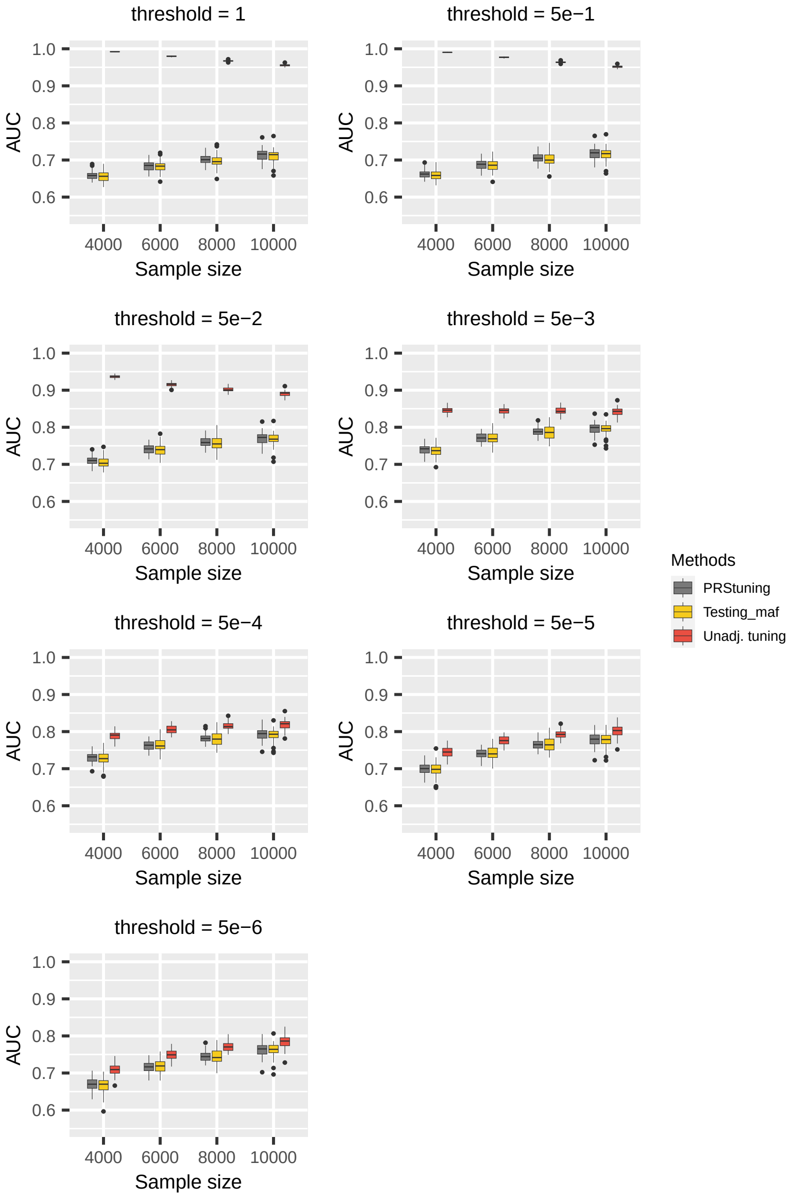

Supplementary Figure 4: AUC boxplots for P+T in the simulation experiments with independent SNPs. The effect sizes of the training and testing data are heterogeneous. Each box represents 50 replications and is presented as median values and the first and third quartiles. The upper/lower whisker extends from the hinge to the largest/smallest value at most 1.5 IQR from the hinge. The grey, yellow, and red panels represent AUC predicted from PRStuning, AUC calculated from testing data, and the unadjusted AUC, respectively. The mean values of ρ_{AUC} and rd_{AUC} for each sample size are respectively $0.963, 0.984, 0.986, 0.987$ and $1.8\%, 1.3\%, 1.2\%, 1.4\%$.

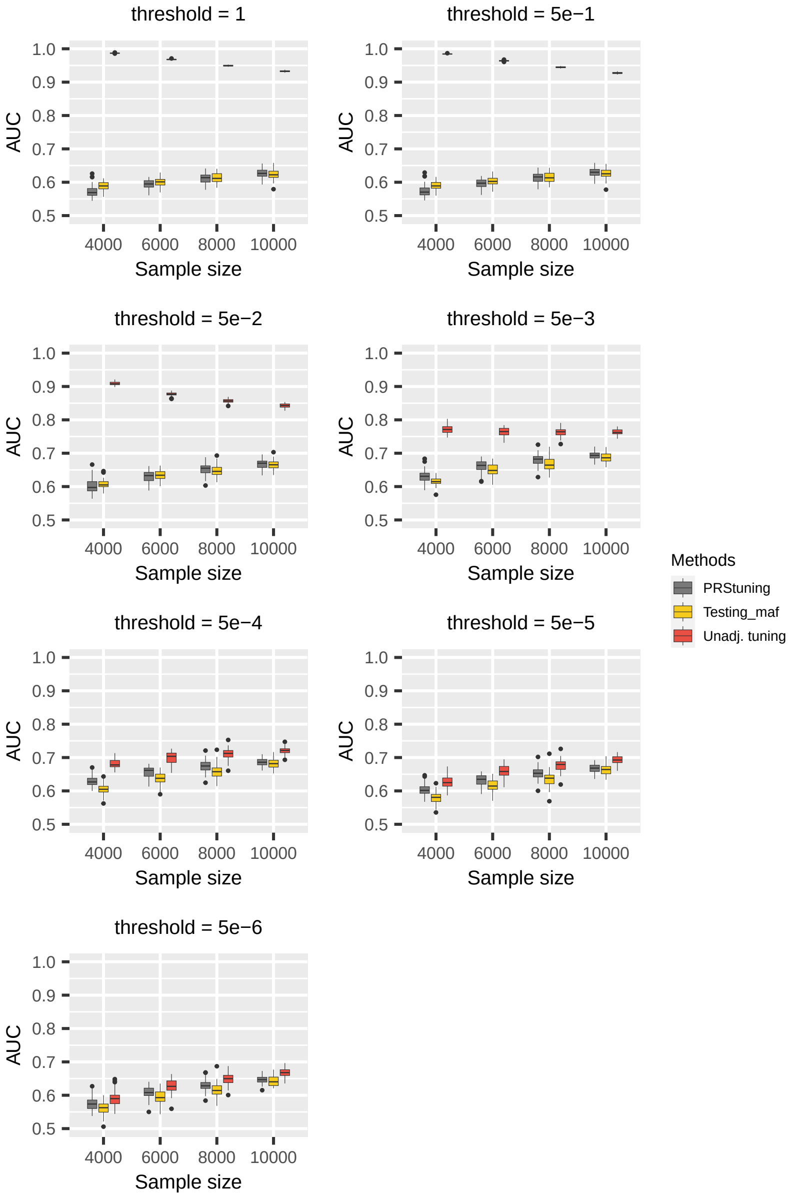

Supplementary Figure 5: AUC boxplots for C+T in simulation experiments with correlated SNPs. The allele frequencies of the training and testing data are heterogeneous. Each box represents 50 replications and is presented as median values and the first and third quartiles. The upper/lower whisker extends from the hinge to the largest/smallest value at most 1.5 IQR from the hinge. The grey, yellow, and red panels represent AUC predicted from PRStuning, AUC calculated from testing data, and the unadjusted AUC, respectively. The mean values of ρ_{AUC} and rd_{AUC} for each sample size are respectively $0.608, 0.849, 0.890, 0.938$ and $3.1\%, 2.4\%, 2.3\%, 1.5\%$.

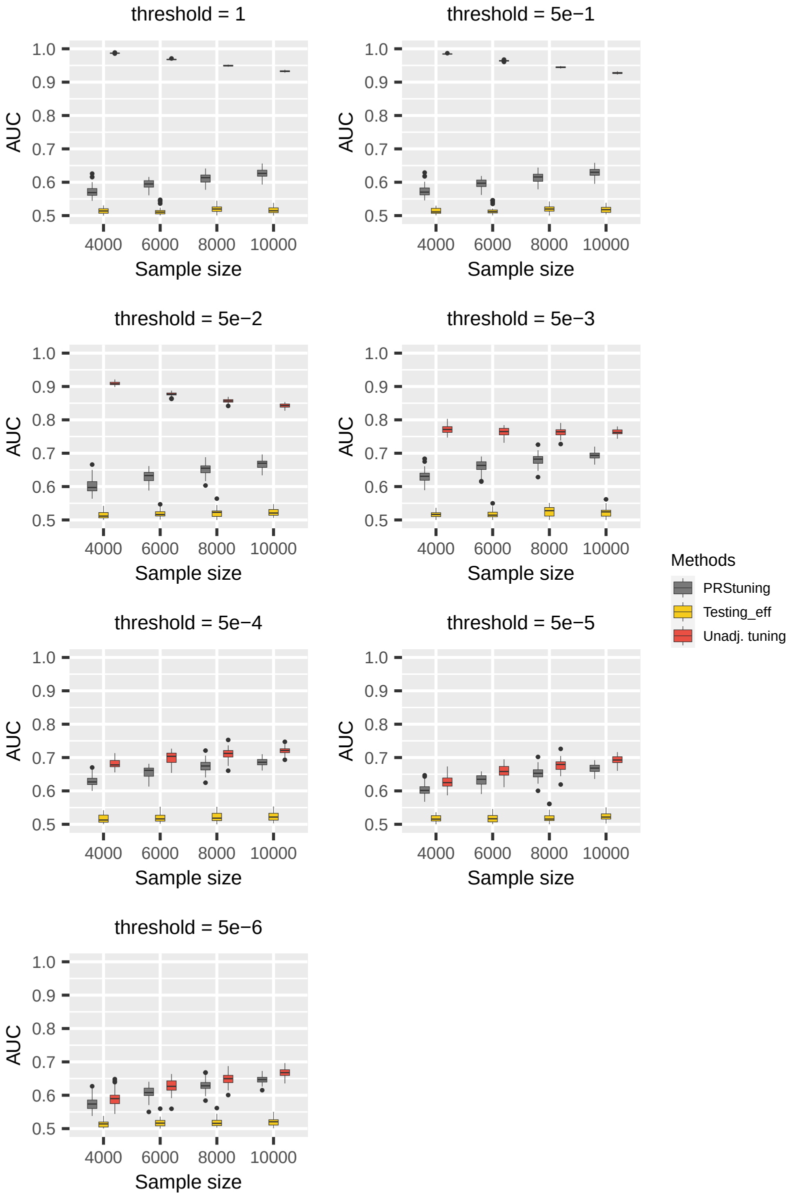

Supplementary Figure 6: AUC boxplots for C+T in simulation experiments with correlated SNPs. The effect sizes of the training and testing data are heterogeneous. Each box represents 50 replications and is presented as median values and the first and third quartiles. The upper/lower whisker extends from the hinge to the largest/smallest value at most 1.5 IQR from the hinge. The grey, yellow, and red panels represent AUC predicted from PRStuning, AUC calculated from testing data, and the unadjusted AUC, respectively. The mean values of ρ_{AUC} and rd_{AUC} for each sample size are respectively $0.022, 0.122, 0.127, 0.272$ and $2.1\%, 2.6\%, 2.8\%, 3.1\%$.

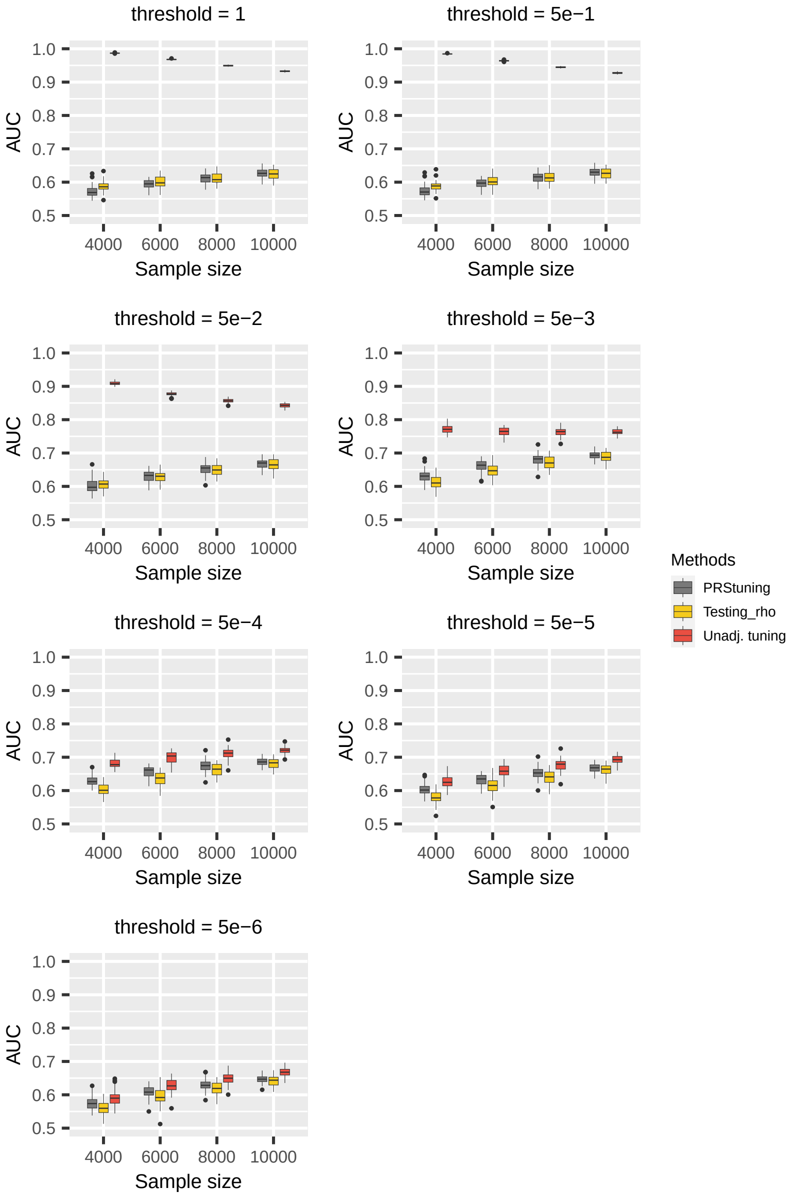

Supplementary Figure 7: AUC boxplots for C+T in simulation experiments with correlated SNPs. The LD structures of the training and testing data are heterogeneous. Each box represents 50 replications and is presented as median values and the first and third quartiles. The upper/lower whisker extends from the hinge to the largest/smallest value at most 1.5 IQR from the hinge. The grey, yellow, and red panels represent AUC predicted from PRStuning, AUC calculated from testing data, and the unadjusted AUC, respectively. The mean values of ρ_{AUC} and rd_{AUC} for each sample size are respectively $0.617, 0.835, 0.922, 0.957$ and $3.3\%, 2.6\%, 1.9\%, 1.5\%$.

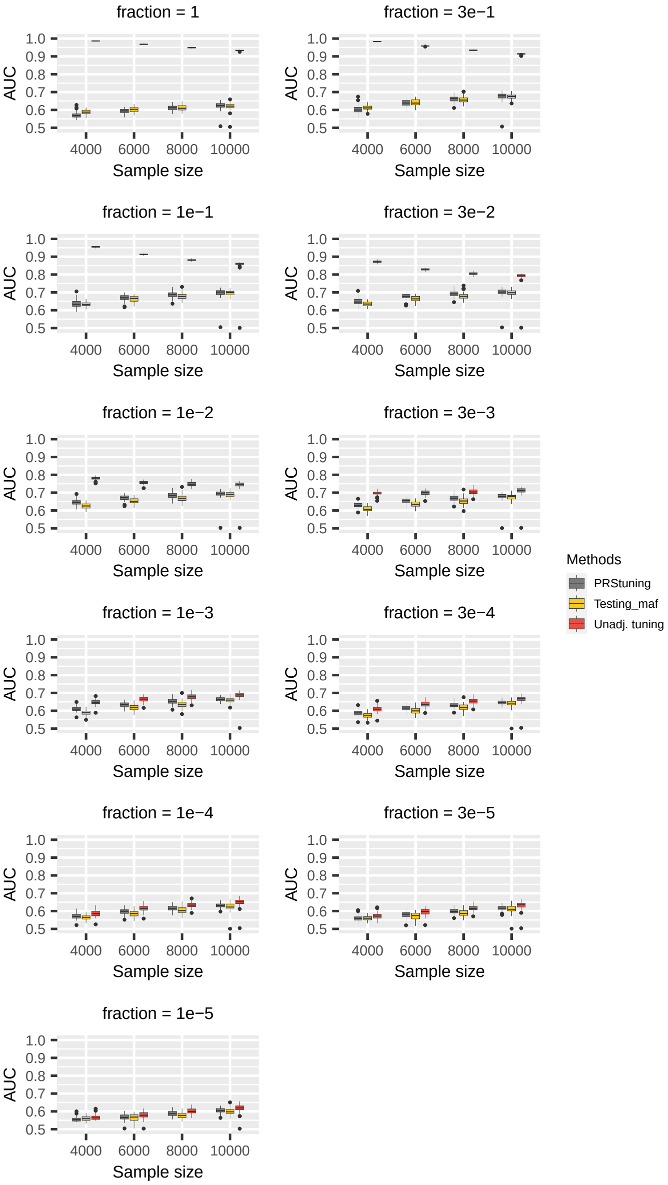

Supplementary Figure 8: AUC boxplots for LDpred in simulation experiments with correlated SNPs. The allele frequencies of the training and testing data are heterogeneous. Each box represents 50 replications and is presented as median values and the first and third quartiles. The upper/lower whisker extends from the hinge to the largest/smallest value at most 1.5 IQR from the hinge. The grey, yellow, and red panels represent AUC predicted from PRStuning, AUC calculated from testing data, and the unadjusted AUC, respectively. The mean values of ρ_{AUC} and rd_{AUC} for each sample size are respectively $0.881, 0.954, 0.966, 0.964$, and $3.0\%, 2.4\%, 2.2\%, 1.5\%$.

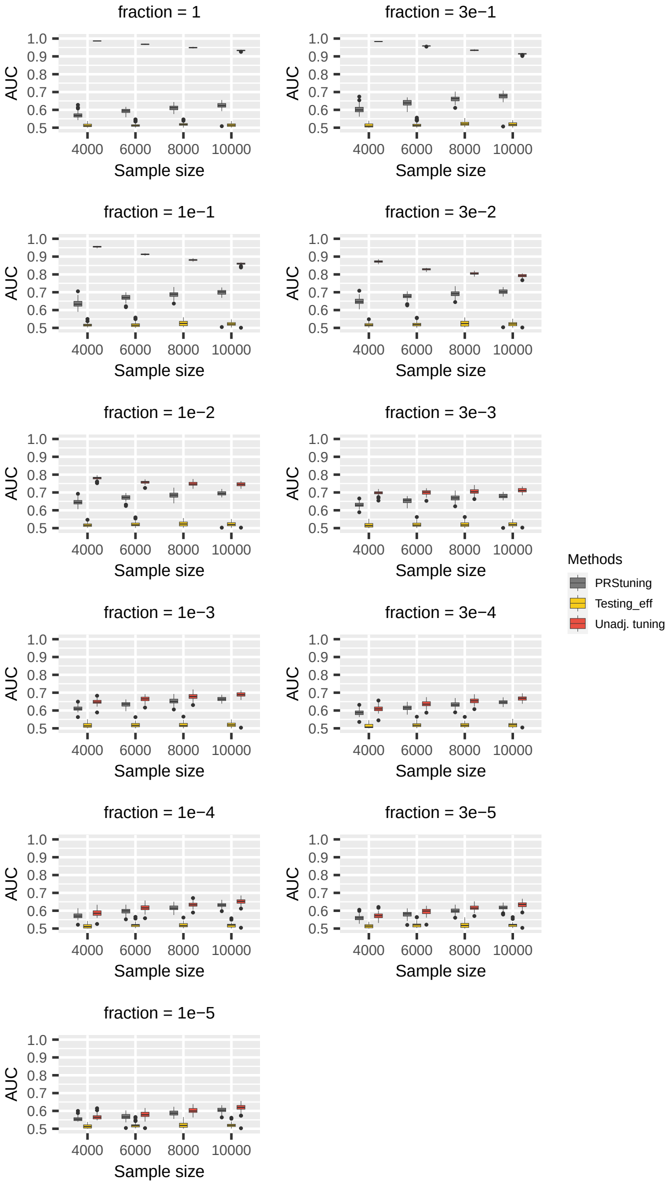

Supplementary Figure 9: AUC boxplots for LDpred in simulation experiments with correlated SNPs. The effect sizes of the training and testing data are heterogeneous. Each box represents 50 replications and is presented as median values and the first and third quartiles. The upper/lower whisker extends from the hinge to the largest/smallest value at most 1.5 IQR from the hinge. The grey, yellow, and red panels represent AUC predicted from PRStuning, AUC calculated from testing data, and the unadjusted AUC, respectively. The mean values of ρ_{AUC} and rd_{AUC} for each sample size are respectively $0.133, 0.056, 0.224, 0.167$ and $2.4\%, 2.8\%, 3.0\%, 3.2\%$.

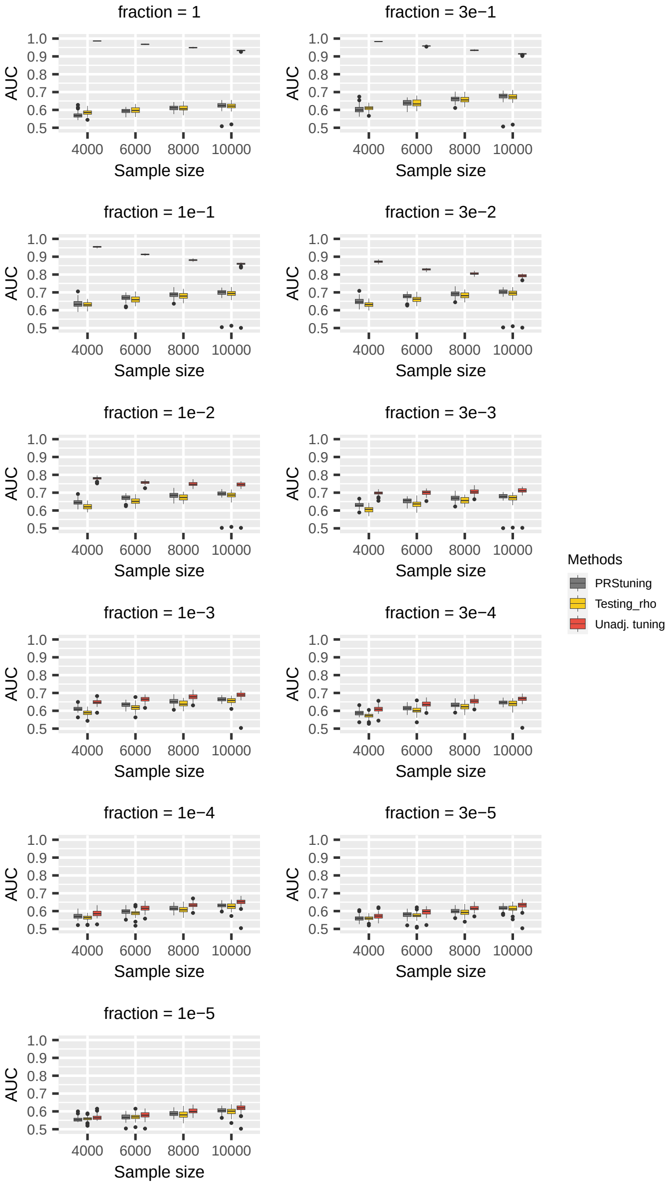

Supplementary Figure 10: AUC boxplots for LDpred in simulation experiments with correlated SNPs. The LD structures of the training and testing data are heterogeneous. Each box represents 50 replications and is presented as median values and the first and third quartiles. The upper/lower whisker extends from the hinge to the largest/smallest value at most 1.5 IQR from the hinge. The grey, yellow, and red panels represent AUC predicted from PRStuning, AUC calculated from testing data, and the unadjusted AUC, respectively. The mean values of ρ_{AUC} and rd_{AUC} for each sample size are respectively $0.871, 0.951, 0.974, 0.976$ and $3.3\%, 2.4\%, 1.8\%, 1.8\%$.

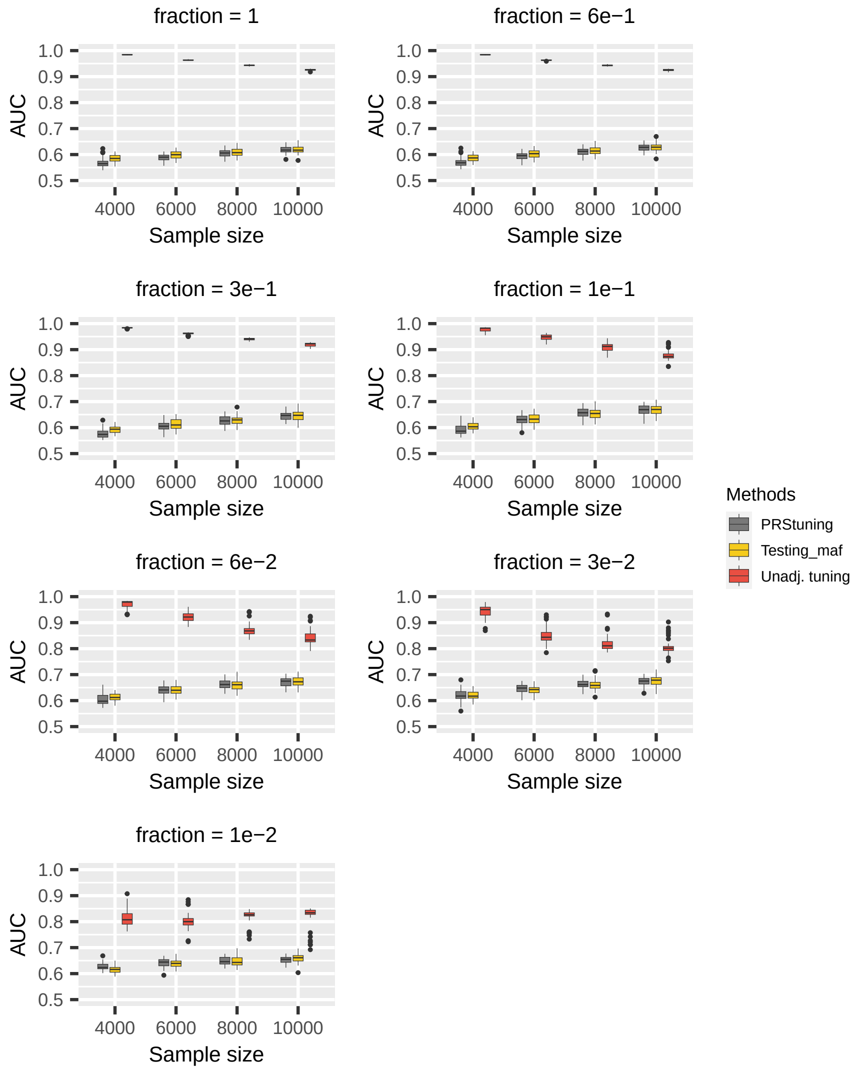

Supplementary Figure 11: AUC boxplots for LDpred2 in simulation experiments with correlated SNPs. The allele frequencies of the training and testing data are heterogeneous. Each box represents 50 replications and is presented as median values and the first and third quartiles. The upper/lower whisker extends from the hinge to the largest/smallest value at most 1.5 IQR from the hinge. The grey, yellow, and red panels represent AUC predicted from PRStuning, AUC calculated from testing data, and the unadjusted AUC, respectively. The mean values of ρ_{AUC} and rd_{AUC} for each sample size are respectively $0.912, 0.970, 0.982, 0.977$ and $2.5\%, 2.0\%, 1.8\%, 1.6\%$.

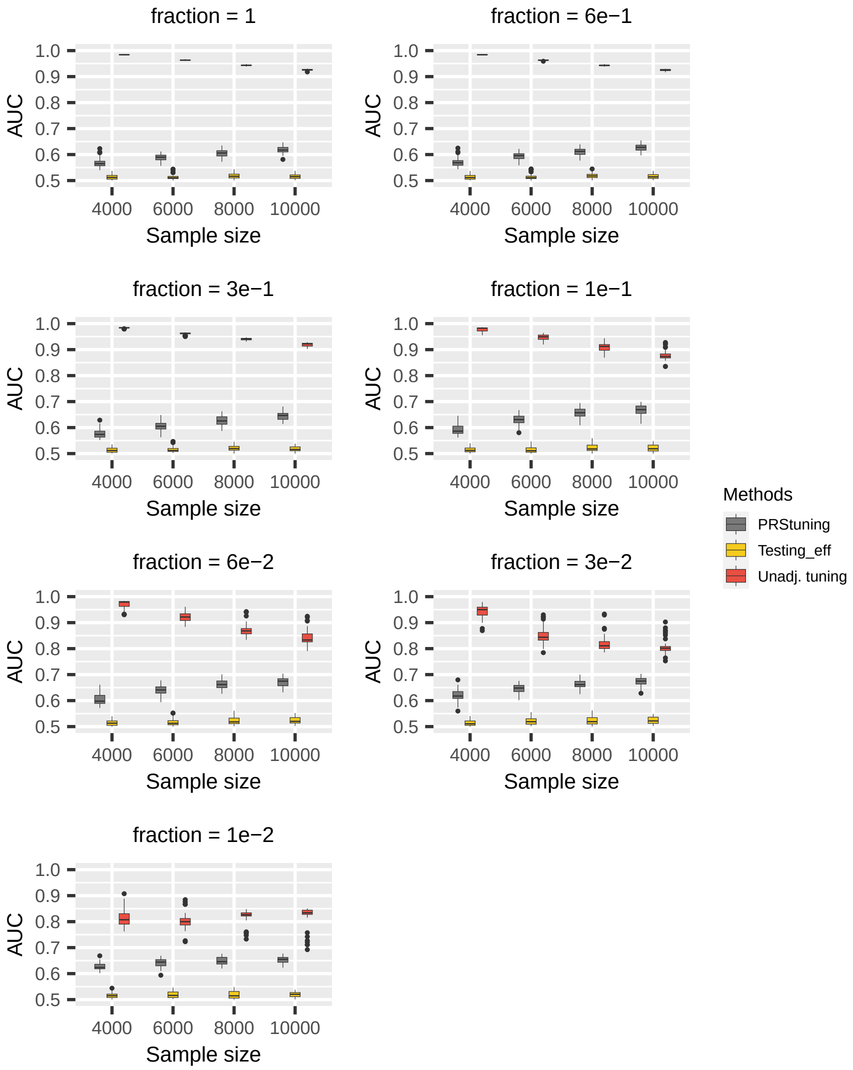

Supplementary Figure 12: AUC boxplots for LDpred2 in simulation experiments with correlated SNPs. The effect sizes of the training and testing data are heterogeneous. Each box represents 50 replications and is presented as median values and the first and third quartiles. The upper/lower whisker extends from the hinge to the largest/smallest value at most 1.5 IQR from the hinge. The grey, yellow, and red panels represent AUC predicted from PRStuning, AUC calculated from testing data, and the unadjusted AUC, respectively. The mean values of ρ_{AUC} and rd_{AUC} for each sample size are respectively $0.137, 0.181, 0.273, 0.390$ and $2.0\%, 2.4\%, 2.7\%, 2.8\%$.

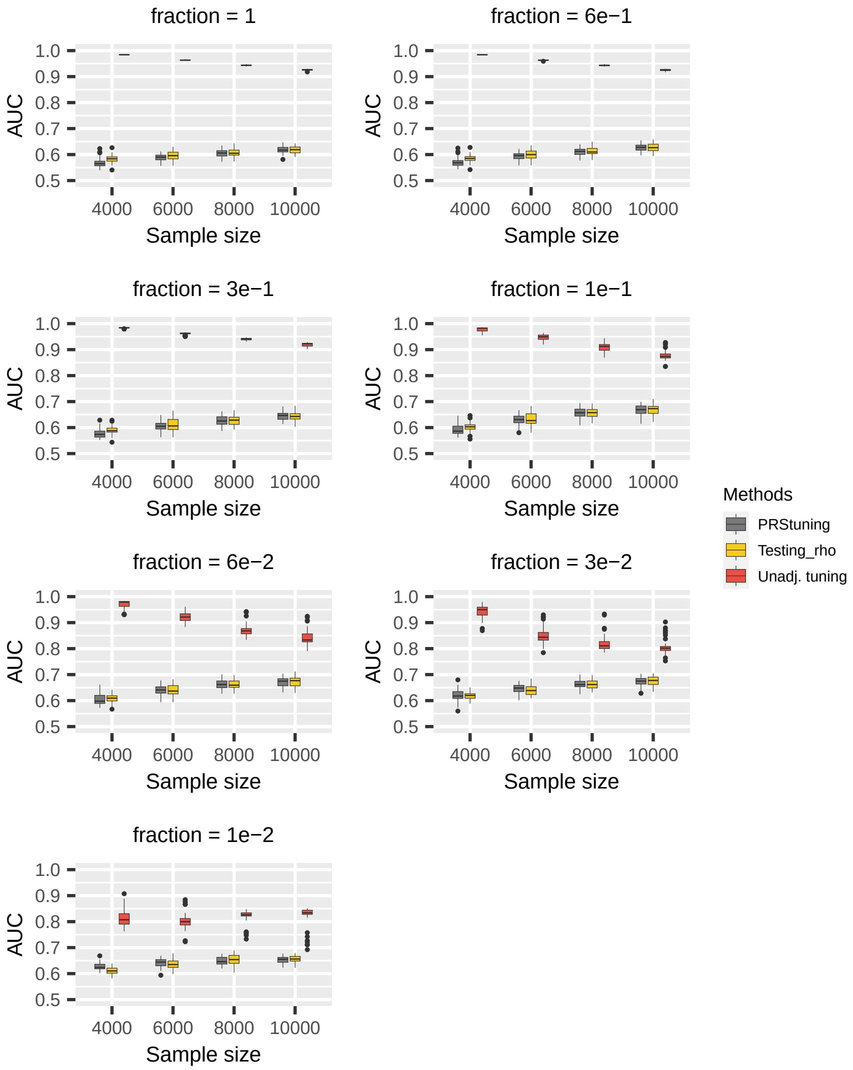

Supplementary Figure 13: AUC boxplots for LDpred2 in simulation experiments with correlated SNPs. The LD structures of the training and testing data are heterogeneous. Each box represents 50 replications and is presented as median values and the first and third quartiles. The upper/lower whisker extends from the hinge to the largest/smallest value at most 1.5 IQR from the hinge. The grey, yellow, and red panels represent AUC predicted from PRStuning, AUC calculated from testing data, and the unadjusted AUC, respectively. The mean values of ρ_{AUC} and rd_{AUC} for each sample size are respectively $0.888, 0.960, 0.974, 0.983$ and $2.4\%, 2.1\%, 1.5\%, 1.8\%$.

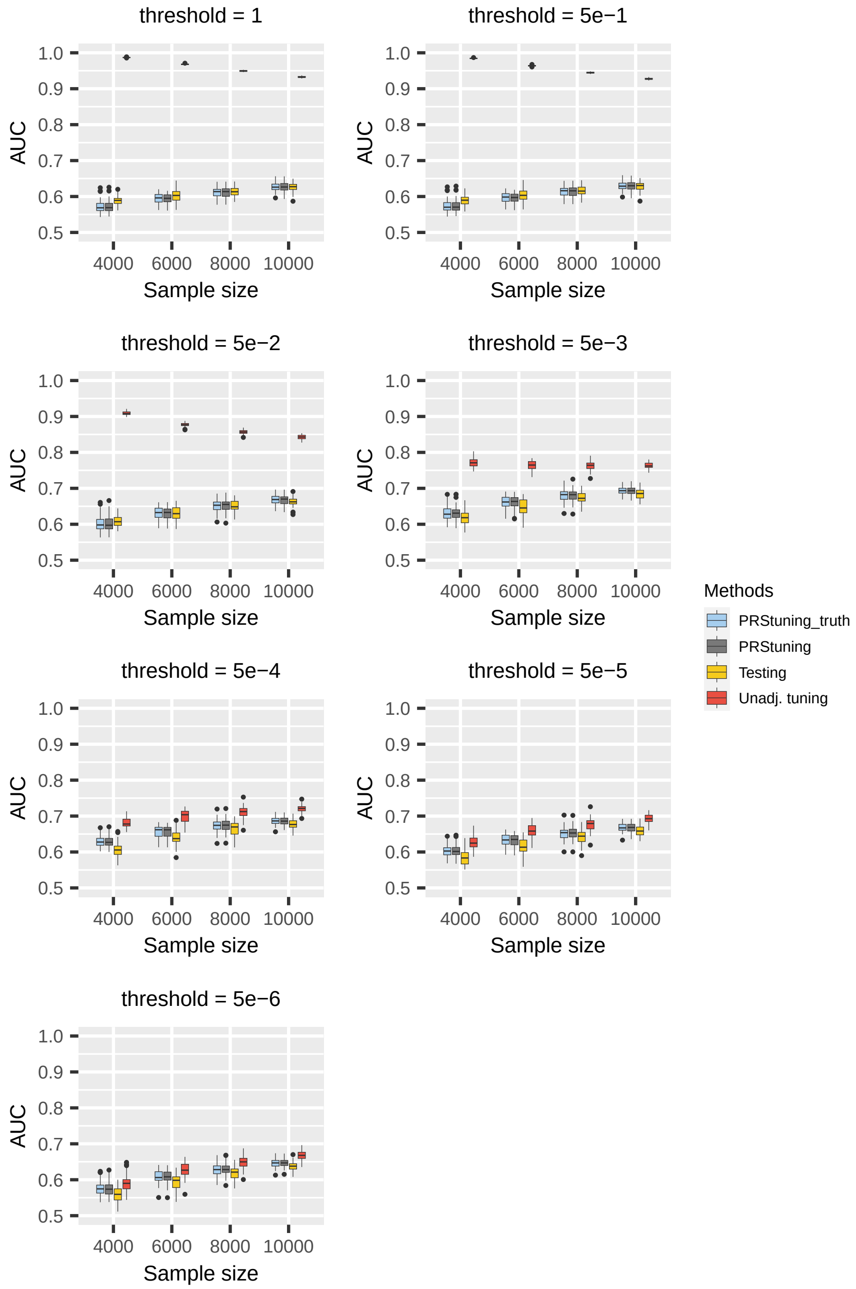

Supplementary Figure 14: AUC boxplots for C+T in simulation experiments with correlated SNPs. Each box represents 50 replications and is presented as median values and the first and third quartiles. The upper/lower whisker extends from the hinge to the largest/smallest value at most 1.5 IQR from the hinge. The blue, grey, yellow, and red panels represent AUC predicted from PRStuning using ground truth LD matrix, AUC predicted from PRStuning, AUC calculated from testing data, and the unadjusted AUC, respectively.

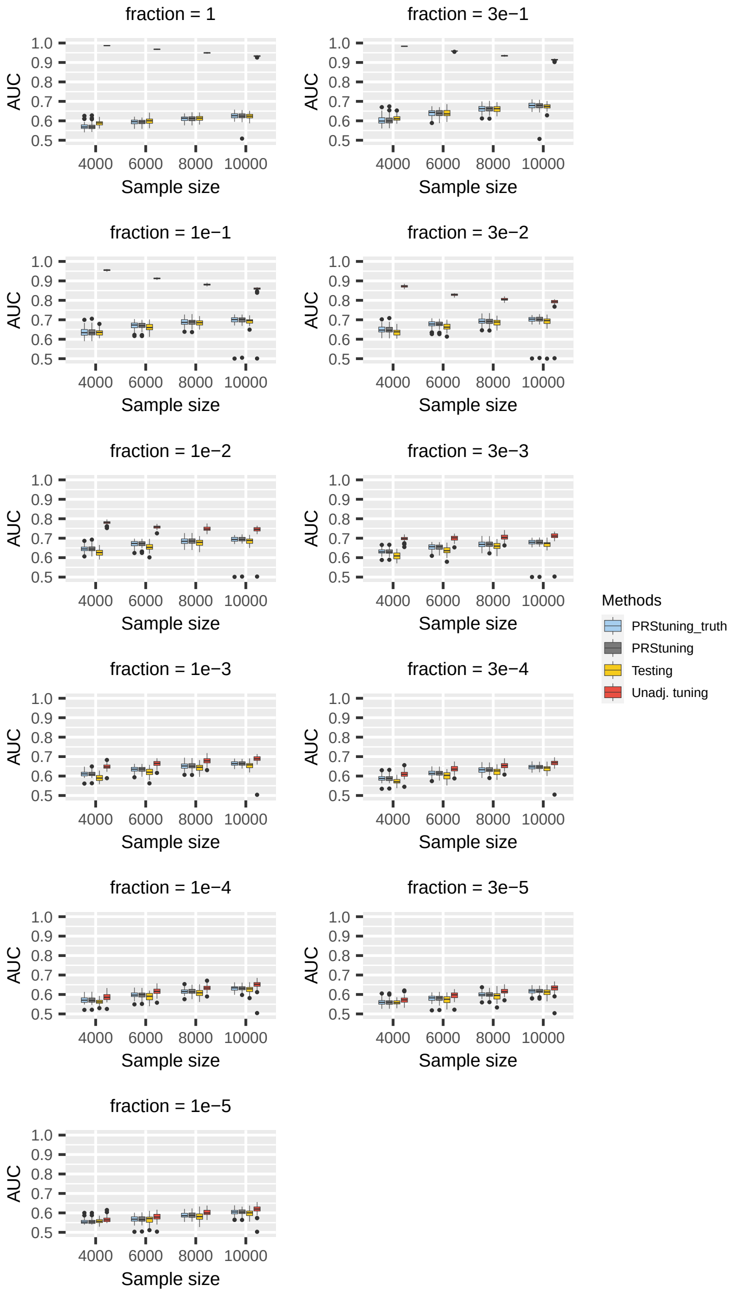

Supplementary Figure 15: AUC boxplots for LDpred in simulation experiments with correlated SNPs. Each box represents 50 replications and is presented as median values and the first and third quartiles. The upper/lower whisker extends from the hinge to the largest/smallest value at most 1.5 IQR from the hinge. The blue, grey, yellow, and red panels represent AUC predicted from PRStuning using ground truth LD matrix, AUC predicted from PRStuning, AUC calculated from testing data, and the unadjusted AUC, respectively.

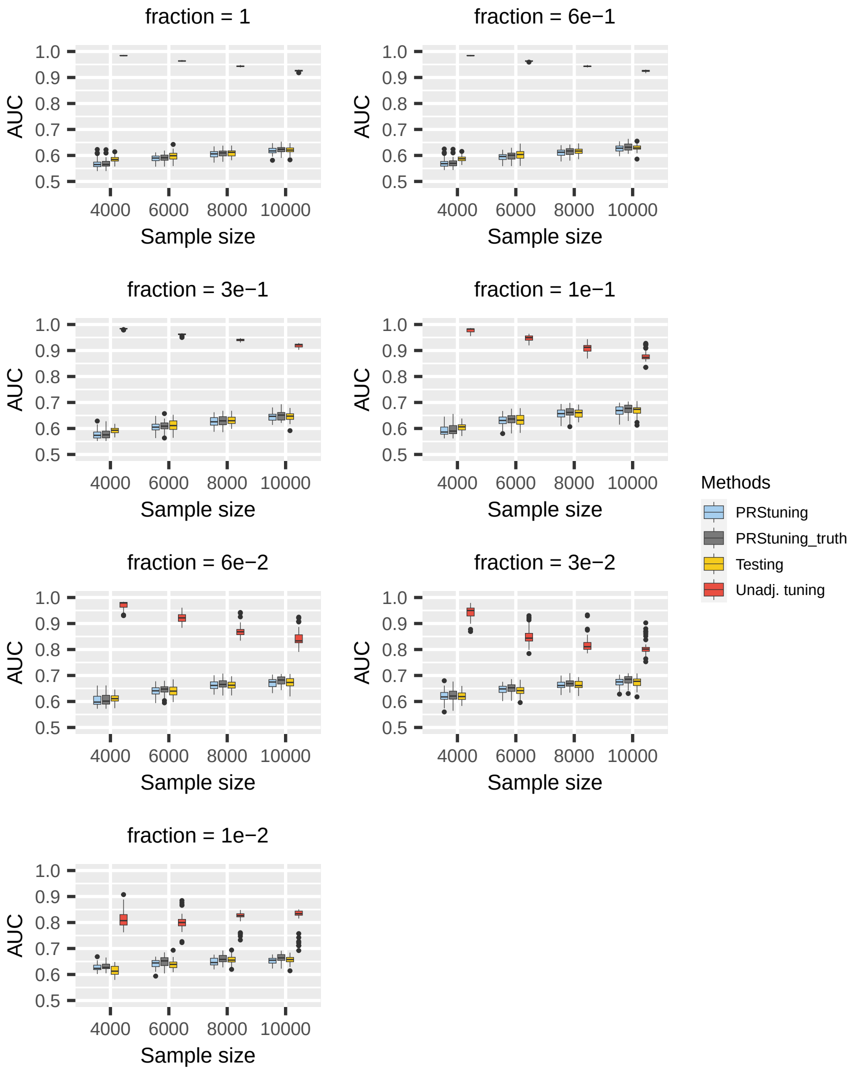

Supplementary Figure 16: AUC boxplots for LDpred2 in simulation experiments with correlated SNPs. Each box represents 50 replications and is presented as median values and the first and third quartiles. The upper/lower whisker extends from the hinge to the largest/smallest value at most 1.5 IQR from the hinge. The blue, grey, yellow, and red panels represent AUC predicted from PRStuning using ground truth LD matrix, AUC predicted from PRStuning, AUC calculated from testing data, and the unadjusted AUC, respectively.

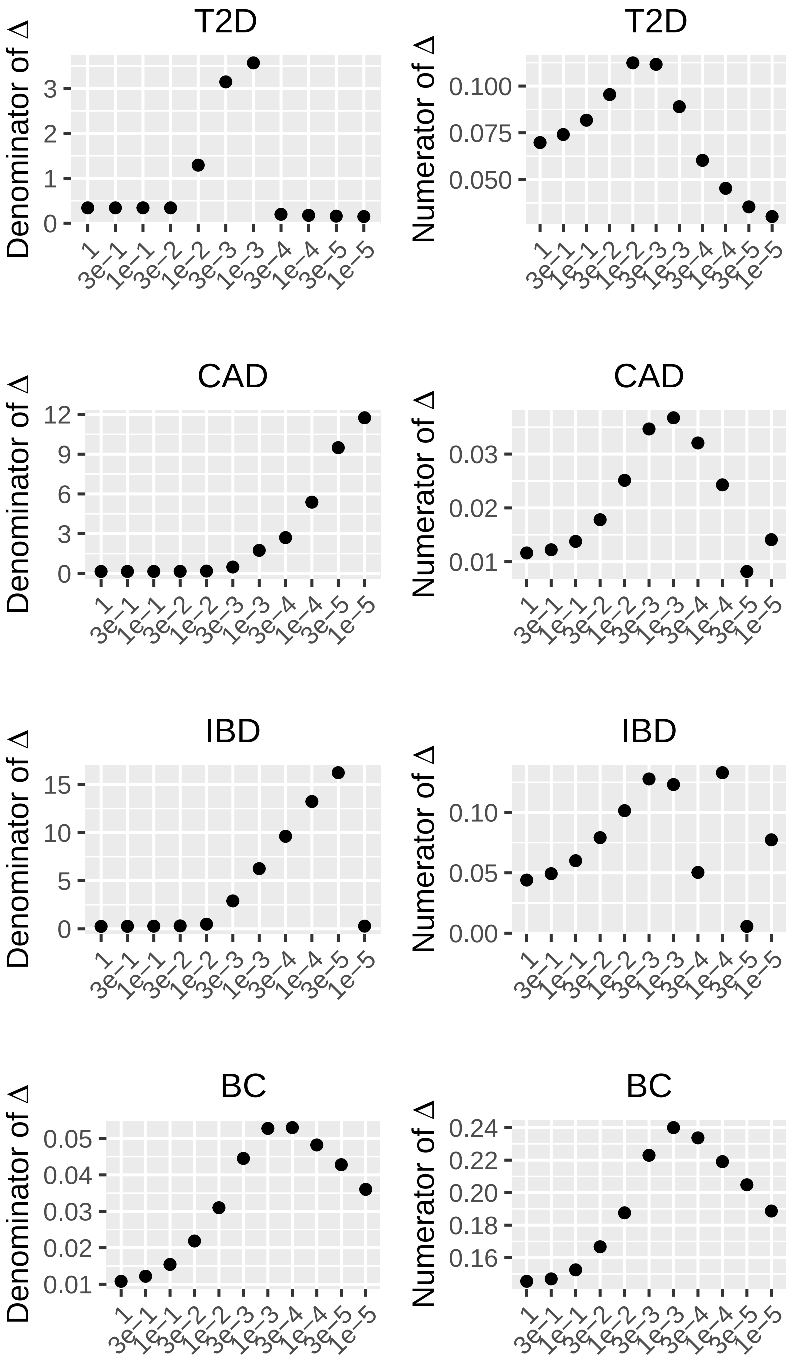

Supplementary Figure 17: The denominators and numerators of Δ based on different parameter values in LDpred. The left panel is the denominators and the right panel is the numerators in Δ calculation. Although both denominators and numerators were unimodal functions with respect to parameter values, their ratios led the Δ to become bimodal functions.

Supplementary References

Agresti, A. (2012). *Categorical data analysis*, volume 792. John Wiley & Sons.

Bishop, C. M. and Nasrabadi, N. M. (2006). *Pattern recognition and machine learning*, volume 4. Springer.

Doucet, A., Godsill, S. J., and Robert, C. P. (2002). Marginal maximum a posteriori estimation using Markov chain Monte Carlo. *Statistics and Computing*, 12(1):77–84.

Lee, S. H., Wray, N. R., Goddard, M. E., and Visscher, P. M. (2011). Estimating missing heritability for disease from genome-wide association studies. *The American Journal of Human Genetics*, 88(3):294–305.
