# Supplementary material for: Tuning parameters for polygenic risk score methods using GWAS summary statistics from training data
Source: Nat Commun. 2024 Jan 2;15:24. doi: 10.1038/s41467-023-44009-0 (PMC10762162; doi:10.1038/s41467-023-44009-0)
Supplement: Supplementary file 3 — Reporting Summary [file 41467_2023_44009_MOESM3_ESM.pdf]

nature portfolio

Corresponding author(s): Wei Jiang, Ling Chen, Matthew J. Girgenti, Hongyu Zhao

Last updated by author(s): Nov 13, 2023

Reporting Summary

Nature Portfolio wishes to improve the reproducibility of the work that we publish. This form provides structure for consistency and transparency in reporting. For further information on Nature Portfolio policies, see our Editorial Policies and the Editorial Policy Checklist.

Statistics

For all statistical analyses, confirm that the following items are present in the figure legend, table legend, main text, or Methods section.

n/a	Confirmed	
☐	☒	The exact sample size (*n*) for each experimental group/condition, given as a discrete number and unit of measurement
☐	☒	A statement on whether measurements were taken from distinct samples or whether the same sample was measured repeatedly
☐	☒	The statistical test(s) used AND whether they are one- or two-sided<br>*Only common tests should be described solely by name; describe more complex techniques in the Methods section.*
☒	☐	A description of all covariates tested
☐	☒	A description of any assumptions or corrections, such as tests of normality and adjustment for multiple comparisons
☐	☒	A full description of the statistical parameters including central tendency (e.g. means) or other basic estimates (e.g. regression coefficient) AND variation (e.g. standard deviation) or associated estimates of uncertainty (e.g. confidence intervals)
☐	☒	For null hypothesis testing, the test statistic (e.g. *F*, *t*, *r*) with confidence intervals, effect sizes, degrees of freedom and *P* value noted<br>*Give P values as exact values whenever suitable.*
☐	☒	For Bayesian analysis, information on the choice of priors and Markov chain Monte Carlo settings
☒	☐	For hierarchical and complex designs, identification of the appropriate level for tests and full reporting of outcomes
☐	☒	Estimates of effect sizes (e.g. Cohen's *d*, Pearson's *r*), indicating how they were calculated

Our web collection on statistics for biologists contains articles on many of the points above.

Software and code

Policy information about availability of computer code

Data collection	No software was used for data collection.
Data analysis	In this paper, we used R-4.1, python-3.8, plink-1.9 [https://www.cog-genomics.org/plink/], ldpred [https://github.com/bvilhjal/ldpred], ldpred2 [https://privefl.github.io/bigsnpr/articles/LDpred2.html], PUMAS [https://github.com/qlu-lab/PUMAS]. The codes for PRStuning are available at https://github.com/lscientific/PRStuning.

For manuscripts utilizing custom algorithms or software that are central to the research but not yet described in published literature, software must be made available to editors and reviewers. We strongly encourage code deposition in a community repository (e.g. GitHub). See the Nature Portfolio guidelines for submitting code & software for further information.

Data

Policy information about availability of data

All manuscripts must include a data availability statement. This statement should provide the following information, where applicable:

- Accession codes, unique identifiers, or web links for publicly available datasets
- A description of any restrictions on data availability
- For clinical datasets or third party data, please ensure that the statement adheres to our policy

The 1000genomes data can be downloaded via https://www.internationalgenome.org/, and the HapMap3 data can be downloaded via https://www.sanger.ac.uk/

resources/downloads/human/hapmap3.html. The UK Biobank (UKBB) data are available under restricted access. Researchers can apply for access at https://www.ukbiobank.ac.uk/enable-your-research/apply-for-access. The Type 2 Diabetes GWAS summary level data available from the DIAGRAM consortium [https://diagram-consortium.org/downloads.html]. The Coronary Artery Disease GWA meta-analysis data are available from the CARDIoGRAMplusC4D Consortium [http://www.cardiogramplusc4d.org/data-downloads/]. The Inflammatory Bowel Disease Disease GWAS summary level data are available from the International Inflammatory Bowel Disease Genetics Consortium (IIBDGC) [https://www.ibdgenetics.org/]. The Breast cancer data are available from the Breast Cancer Association Consortium [https://bcac.ccge.medschl.cam.ac.uk/bcacdata/oncoarray/oncoarray-and-combined-summary-result/gwas-summary-results-breast-cancer-risk-2017]. We provide example data for demonstrating the usage of our method at https://github.com/lscientific/PRStuning, where the reference panel and corresponding LD matrix based on the 1000 Genomes Project can also be found. Source data are provided with this paper.

Research involving human participants, their data, or biological material

Policy information about studies with human participants or human data. See also policy information about sex, gender (identity/presentation), and sexual orientation and race, ethnicity and racism.

Reporting on sex and gender	Not applicable
Reporting on race, ethnicity, or other socially relevant groupings	Not applicable
Population characteristics	Not applicable
Recruitment	Not applicable
Ethics oversight	Not applicable

Note that full information on the approval of the study protocol must also be provided in the manuscript.

Field-specific reporting

Please select the one below that is the best fit for your research. If you are not sure, read the appropriate sections before making your selection.

☒ Life sciences ☐ Behavioural & social sciences ☐ Ecological, evolutionary & environmental sciences

For a reference copy of the document with all sections, see nature.com/documents/nr-reporting-summary-flat.pdf

Life sciences study design

All studies must disclose on these points even when the disclosure is negative.

Sample size	In the simulation based on real UKBB genotype data, 1,027,699 SNPs and 272,751 individuals were used.<br>For the T2D dataset, 56,962 controls, 12,171 cases and 718,340 SNPs were used. For the CAD dataset, 64,762 controls, 22,233 cases and 861,825 SNPs were used. For the IBD dataset, 38,155 controls, 48,485 cases and 952,376 SNPs were used. For the BC dataset, 17,588 controls, 14,910 cases and 11, 050, 495 SNPs were used. The sample sizes were determined by quality control preprocessing and overlapping UKBB, 1000 Genomes, HapMap3 datasets. The sample sizes are sufficient compared to most of the datasets used in the current literature.
Data exclusions	Partial data for the UK Biobank data were excluded for quality control purposes. The SNPs with minor allele frequencies <= 5% and genotype-missing rates >=5% were excluded. The SNPs with p-values <= 1 × 10−4 in the Hardy-Weinberg equilibrium tests were excluded. he SNPs with imputed information score INFO <= 0.8 were excluded. The individuals with genotype-missing rate >=1% were excluded. We only selected The selected individuals who reported themselves as white British, and the selected individuals have no family relationships among them.<br>In our method, we chose the 1000 Genomes Project as our default reference panel since it has a larger sample size. Most PRS methods calculate weights on the SNPs genotyped in the HapMap 3 project because it constitutes a set of commonly used tag SNPs that are usually well-imputed in different GWAS. To extract reliable results of the LD matrix and to reduce the computational cost, we only included HM3 SNPs in the reference panel in our experiments. Only the SNPs overlapped between GWAS summary statistics and the testing data were considered in our analyses. This is to ensure the coherence between datasets to allow PRStuning to work effectively.
Replication	Seeds are used in simulation and real data experiments when there is randomness involved. Replications were successful.
Randomization	In simulations, all data allocations were performed randomly. In the simulation based on real genotype data (UKBB), we uniformly randomly sampled 80% individuals among the 272,751 individuals to be the training data and the remaining as testing data.
Blinding	No blinding since this study does not involve estimating treatment effect. The purpose of this study is to evaluate PRS models based on GWAS summary statistics from the training data.

Reporting for specific materials, systems and methods

We require information from authors about some types of materials, experimental systems and methods used in many studies. Here, indicate whether each material, system or method listed is relevant to your study. If you are not sure if a list item applies to your research, read the appropriate section before selecting a response.

Materials & experimental systems

n/a	Involved in the study
☒	☐ Antibodies
☒	☐ Eukaryotic cell lines
☒	☐ Palaeontology and archaeology
☒	☐ Animals and other organisms
☒	☐ Clinical data
☒	☐ Dual use research of concern
☒	☐ Plants

Methods

n/a	Involved in the study
☒	☐ ChIP-seq
☒	☐ Flow cytometry
☒	☐ MRI-based neuroimaging

Plants

Seed stocks	*Report on the source of all seed stocks or other plant material used. If applicable, state the seed stock centre and catalogue number. If plant specimens were collected from the field, describe the collection location, date and sampling procedures.*
Novel plant genotypes	*Describe the methods by which all novel plant genotypes were produced. This includes those generated by transgenic approaches, gene editing, chemical/radiation-based mutagenesis and hybridization. For transgenic lines, describe the transformation method, the number of independent lines analyzed and the generation upon which experiments were performed. For gene-edited lines, describe the editor used, the endogenous sequence targeted for editing, the targeting guide RNA sequence (if applicable) and how the editor was applied.*
Authentication	*Describe any authentication procedures for each seed stock used or novel genotype generated. Describe any experiments used to assess the effect of a mutation and, where applicable, how potential secondary effects (e.g. second site T-DNA insertions, mosiacism, off-target gene editing) were examined.*
